# A Methodology for the Design of Application-Specific Cyber-Physical Social Sensing Co-Simulators

**DOI:** 10.3390/s17102177

**Published:** 2017-09-22

**Authors:** Borja Bordel Sánchez, Ramón Alcarria, Álvaro Sánchez-Picot, Diego Sánchez-de-Rivera

**Affiliations:** 1 Department of Telematics Systems Engineering, Universidad Politécnica de Madrid, Avenida Complutense n° 30, 28040 Madrid (España), Spain; alvaro.spicot@gmail.com (Á.S.-P.); diegosanchez@dit.upm.es (D.S.-d.-R.); 2 Department of Topographic Engineering and Cartography, Universidad Politécnica de Madrid, Campus Sur, 28031 Madrid (España), Spain; ramon.alcarria@upm.es

**Keywords:** Cyber-Physical Social Sensing, co-simulation, social simulation, networks simulators, MASON, NS3, Cyber-Physical Systems

## Abstract

Cyber-Physical Social Sensing (CPSS) is a new trend in the context of pervasive sensing. In these new systems, various domains coexist in time, evolve together and influence each other. Thus, application-specific tools are necessary for specifying and validating designs and simulating systems. However, nowadays, different tools are employed to simulate each domain independently. Mainly, the cause of the lack of co-simulation instruments to simulate all domains together is the extreme difficulty of combining and synchronizing various tools. In order to reduce that difficulty, an adequate architecture for the final co-simulator must be selected. Therefore, in this paper the authors investigate and propose a methodology for the design of CPSS co-simulation tools. The paper describes the four steps that software architects should follow in order to design the most adequate co-simulator for a certain application, considering the final users’ needs and requirements and various additional factors such as the development team’s experience. Moreover, the first practical use case of the proposed methodology is provided. An experimental validation is also included in order to evaluate the performing of the proposed co-simulator and to determine the correctness of the proposal.

## 1. Introduction

**Figure 1:**
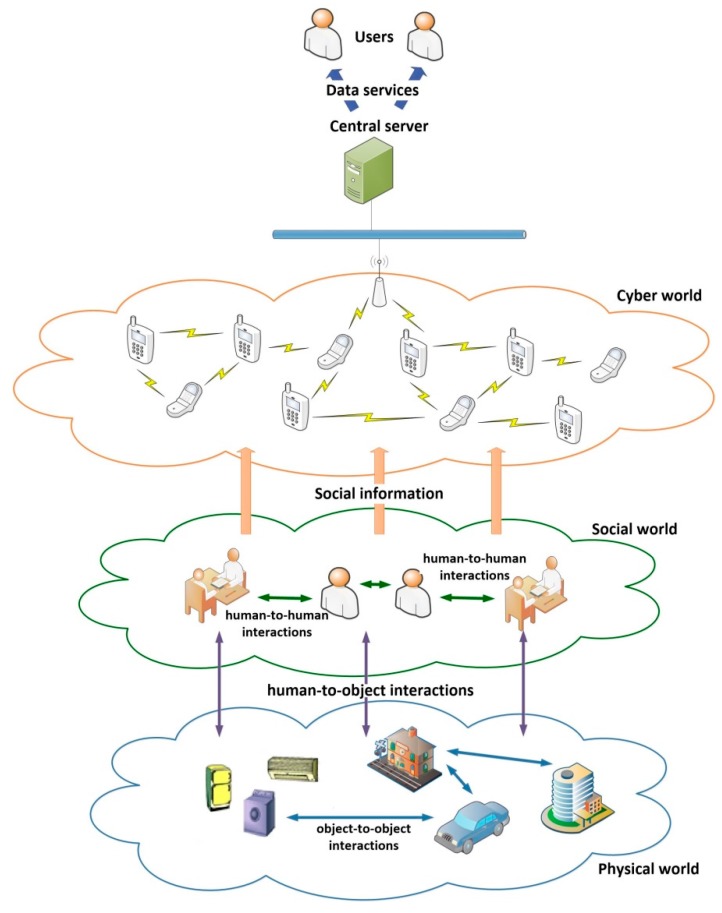
Cyber-Physical Social Sensing (CPSS) scheme.

Cyber-Physical Social Sensing (CPSS) systems [1] are ubiquitous mobile wireless sensor networks where intelligent terminals equipped with various sensors are integrated. These terminals perceive human social information such as the environment or the social activities. CPSS include also a central server where the information is uploaded and from which users are provided with data services (see Figure 1). The final objective of CPSS is to enrich human-to-human, human-to-object, and object-to-object interactions in the physical world, human society, as well as in the virtual world [1].

As can be deducted from the previous description, CPSS are intrinsically concurrent. Various domains (such as the social, physical or cyber) coexist in time, evolve together and influence each other [2] (see Figure 1).

In CPSS, the social world includes all people sharing the same space (as large as wanted) and time, including their individual and group physical and physiological state and all human-to-human interactions among the habitants (affective or physical) [3,4]. Many specialized works on sociology and group psychology have studied these issues [5,6,7,8].

The physical world includes all physical phenomena and third-party devices and objects which are relevant to the CPSS operation [9]. The physical world also includes all the processes and object-to-object interactions developed by the elements which compose it. The temperature evolution in closed rooms [10], or the traditional industrial systems [11], belong to the physical world. The impact of the physical world in the social world has been exhaustively studied in the context of the so-called social environment [12,13,14,15], but always from a narrative point of view (never with practical applications).

Finally, the cyber world includes all the intelligent terminals [16], sensors and mobile wireless networks used to sense the social information. The relation between the cyber world and the physical world is probably the most studied. The term cyber-physical systems (CPS) [17] (which refers to the intersection of the physical and cyber worlds) is nowadays one of the most popular terms in research. On the other hand, the field of social sensing [18] (the union of the cyber and social worlds) are also turning more important day by day (with concepts such as the Social Internet-of-Things [18,19]), although it continues to be less popular than CPS.

The novelty of CPSS, then, is to consider the three subsystems (sometimes together with other domains such as the mental world [20]) evolving together, formalizing some ideas which have started to appear very recently (such as the union of CPS and social sensing) [21]. Table 1 summarizes the relations among the different subsystems as described above.

As a current technological paradigm, CPSS present some common characteristics with other recent proposals such as the Internet-of-Things (IoT). However, while IoT solutions are focused on the global interconnection of “things” (daily living objects provided with embedded computers), CPSS are primary designed to perceive human social information including the environment, transportation and social activities [22]. In both cases, a heterogeneous hardware platform with a high density of devices is considered. Nevertheless, IoT systems consider people as users and things as the main element in the systems, while in CPSS the human factor is the basic information source and sensors (usually integrated into intelligent devices) are only the medium through which obtain that information. Technically, CPSS may be understood as an extension of IoT systems, were human factors are combined with traditional devices. As an evolution of Cyber-Physical Systems [23], CPSS use to define feedback control loops, where social information is processed and returned to social world in order to generate a certain effect. On the other hand, IoT services do not have restrictions and may be based on the simple data acquisition.

In any case, it must be noted that how the social world is integrated into CPS/IoT in order to create CPSS systems is an open issue [20]. Some works (the oldest ones) monitors social information by means of user surveys or user interfaces [24], defining kind of collaborative platforms (in order to forecast the bus arrivals [25] for example). Other define sensor networks were nodes interact with others and share information as people do in social networks [26]. Finally, and probably the most popular approach nowadays, some authors understand that CPSS should monitor people in a passive way (without human intervention, surveys or interfaces) as CPS monitors temperature or manufacturing process. In order to do that, sensors included in intelligent terminals are usually employed [27,28]. Using the acquired information, people behavior, movement, etc. could be tracked and the environment (public infrastructure, such as airports, for example) would react to this social information.

In this work, we are following the last approach, considering that CPSS are systems focused on monitoring people social behavior in a passive, unobtrusive and non-intrusive way.

The most important difficulty that CPSS designers face is the problem of constructing large systems, including thousands of devices, people and a great amount of information [29]. Besides, CPSS have to manage very different abstraction levels, from the high-level information typical of the social world, to the very low-level information, so common in the cyber world (especially when hardware is considered) [20]. In order to address this problem, application-specific tools are necessary for specifying and validating designs and simulating systems.

Nowadays, specific tools are employed to simulate each domain independently (a social simulator for the social world, a network simulator for the cyber world, etc.). This solution is very efficient in time, as each tool is extremely optimized to simulate a specific domain. In fact, most new tools for CPSS simulation would be less efficient and would require more computing power [30]. However, this situation also presents important disadvantages. Mainly, it firstly forces the decomposition of the CPSS scenario in different subsystems, simulating each domain in one environment and, later, relates the results obtained from each tool. All these actions must be performed by different experts, simulations becoming a very costly and complicated task. Thus, despite the probable inefficiency in time, the future is the co-simulation [31].

Nevertheless, today there is a lack of co-simulation instruments being able to simulate together all domains which made up a CPSS. The main cause of this situation is the difficulty of relating correctly the simulation of various subsystems, as well as the complexity of interaction with final users in order to acquire the necessary co-simulator’s requirements and characteristics. In particular, integrating and/or synchronizing various simulation tools implies modifying the execution routine or the communication and graphic interfaces among other tasks. Moreover, interacting with final users in order to acquire the required system characteristics implies to coordinate a communication process among all the stakeholders. Most of these activities are very time consuming and many times researches, companies or users do not implement new co-simulation tools for not being sure whether the effort is worthwhile. In that situation, the role of software architects is vital, as they must guarantee that the selected design or solution is the most adequate for every case: depending on the application, the users’ needs, the acquired requirements, the development team and the simulations to be performed (among other topics). In order to obtain a truly correct, efficient and functional co-simulator, appropriate to each specific situation, all important factors must be considered following an ordered process. However, the methodology that software architects should follow in order to acquire the users’ requirements and select their own design for an application-specific co-simulator has been scarcely studied: only partial and incomplete proposals have been reported.

Therefore, in this paper a methodology for the design of CPSS co-simulation tools is proposed. The work describes the four steps that software architects should follow in order to design their own application-specific CPSS co-simulator, as well as the previous phase of requirements and characteristics capture involving both software architects and final users. The proposal includes from the characteristics fixing and the selection of the most appropriate co-simulation paradigm, to the choice of the graphic environments for results presentation. The paper provides, also, the first practical use case of the proposed methodology, implementing the obtained design using currently available technologies. Finally, results obtained from the implemented CPSS co-simulator are included. From the analysis of these experimental results, future challenges and research lines are identified.

During the last months CPSS has become a very popular research topic. Different works about the applications of the CPSS paradigm to social Big Data [32], robotics [26], people tracking [27] and wireless sensor networks (WSN) [33] have been recently reported. In most of these papers, experimental validations are based on laboratory deployments, theoretical analyses or concept proofs [24]. However, some variables (such as scalability) are not easily evaluable using these techniques and/or imply a huge cost. Thus, papers consisting of an exhaustive evaluation of algorithms for CPSS or other similar proposals usually include the design and implementation of a CPSS co-simulator [34,35]. A commercial co-simulator may require several months or years before it can be released, however, many times, CPSS researchers need to design and built and ad-hoc co-simulator in order to evaluate the performance of their proposals. The scope of these instruments is to be used as a general-purpose CPSS co-simulator, but be employed in a specific application. In that way, the objective of this paper is to guide every researcher working on CPSS (which sometimes does not have technical knowledge on programming or simulator construction) during the design process of his own application-specific co-simulator.

The rest of the paper is organized as follows. Section 2 reviews previous works about methodologies for co-simulation tools design and describes the state of the art in social, physical and cyber simulation. Section 3 describes the proposed methodology and discusses the requirements all CPSS co-simulator should fulfill. Later it deeply discusses the four steps which made up the proposed methodology, including the requirements capture and characteristic fixing, the different available co-simulation paradigms, the simulation model and simulation lifecycle, the different coordination and synchronization mechanisms, and the different options for the graphic environment for results presentation. Section 4 describes the first use case of the proposed methodology and the proposed experimental validation in order to evaluate its performance and the correctness of the proposed methodology. Finally, Section 5 shows the results obtained from the experiments. Section 6 concludes the paper and refers some future challenges.

## 2. State of the Art

In this section, a general overview about the recent proposals on CPSS is provided. Moreover (in the second subsection), most relevant works on co-simulation are revised. In the third subsection, specific proposals about CPSS co-simulation are reviewed. Finally, current tools and proposals for simulating every domain involved in CPSS are revised (first physical processes simulators, secondly social simulators and finally network simulator).

### 2.1. Recent Proposals on CPSS

CPSS is an acronym which may refer various proposals: from Cyber-Physical Social Sensing to Cyber-Physical Social Systems [36]. Furthermore, it is not clear if all these proposals refer to the same reality or present some differences [22]. However, in both cases, CPSS represents the deep integration of traditional technological systems (such as the Internet of Things or Cyber-Physical Systems) with human and social factors, such as people behavior, social opinions or the user mental world [20].

In that way, there is not a unique manner to understand Cyber-Physical Social Sensing. Some works reviewing the different approaches may be found [37].

Some proposals understand that, in CPSS, traditional sensors (present in IoT/CPS scenarios) are substituted by user surveys or user interfaces, which are displayed on mobile smart devices [24]. In that way, people opinion is sensed, creating a kind of collaborative of participative platform for relevant data sharing, in the same way as current social networks [38]. Historically, this approach is, probably, the first one to appear. With this view, systems to predict the bus arrivals [25] or to share information among people in the context of smart cities [39] have been reported. Even, in this kind of proposals, it is very necessary to design (and have been proposed) techniques to protect user personal information (as collaborative platforms require creating a user profile) [40]. Methodologies to design CPSS in an effective way have been also reported [41]. In relation to the promising Industry 4.0 paradigm [42], proposals about new manufacturing paradigms including social networks, social businesses and other similar technologies may be found [43].

On the other hand, other works defend the proposal that CPSS are made of traditional sensors, but being able to stablish ad hoc connections in order to share relevant information with other nodes, as people do in social networks. Proposals related to social robots [26], social techniques for information dissemination in sensor networks [44], social ad hoc networks [45] and social routing protocols [46,47] have been reported around this topic.

Finally, some works describe CPSS, but deal with the problem of sensing the human behavior in a passive and non-intrusive way. It is probably the most recent and popular approach nowadays. In relation to Industry 4.0, the concept of social sensor has been proposed [48]. It consists of a virtual entity acquiring information and statistics in a proactive way from the applications and sensors in the user smartphones. Moreover, works about privacy protection, in order to anonymize the acquired data from users are also common [49,50]. Sampling algorithms in order to collect information from users in an unobtrusive way have been also proposed [51]. Frameworks and architectures focused on collecting and processing information about human behavior [27,28] may be also found. Service composition techniques [36], efficient wireless data transmission [52], etc., for CPSS following this last approach have been also investigated.

### 2.2. State-of-the Art on Co-Simulation

The term co-simulation may be understood in various ways. However, for this work, we are following Broman’s proposal, which defines co-simulation as any simulation of a coupled system performed through the composition of various simulators [53]. However, contrary to these previous proposals focused on the coordination of tools considered as black boxes, in this work domain specific tools could be slight modified if required and possible.

Traditional works on co-simulation investigate the coordination between simulators with different time representations (discrete time and continuous time simulators, an approach usually named as hybrid co-simulation) [30]. Problems such as the multi-level modeling (reality may be modeled in different ways depending on the abstraction level) [54] or the stability of dynamical systems [55] have been also addressed.

In relation to the coordination of different simulation tools various standard technologies have been proposed. Basically, three of them are the most important: High-level architecture (HLA) [56], Functional Mock-up Interface (FMI) [57] and Discrete Event System Specification (DEVS) [58].

HLA standard is mainly focused on continuous time (CT) simulations. This standard defines an interface, an object model and a set of rules in order to allow simulation tools to communicate regardless the underlying platform. FMI, on the contrary, is usually employed with discrete-event oriented simulations. This standard was specifically designed to simulate Cyber-Physical Systems, and it is based on the creation of a simulation model or a software library called Functional Mock-up Unit (FMU). Finally, DEVS is also focused on discrete systems, although it is different from the previously mentioned proposal as it is a formalized rather than an available technology.

CPSS co-simulation is natively hybrid. Social and physical simulations are (in general) continuous in time, while cyber (network) simulations are based on discrete events. In that way, proposed standards are not completely useful as, as indicated by Broman [53], both standards have limitations for hybrid co-simulation. In fact, some extension of these standards for certain applications have been proposed [59,60], although these proposals do not totally resolve the problem.

### 2.3. CPSS Co-Simulation

A CPSS simulator should be able to simulate all the subsystems composing a CPSS. That implies not only representing elements belonging to each domain, but also considering specific and precise models for them. For example, as we are seeing, various social simulators allow users to include smartphones in simulations, however, no model for the cellular network, congestion, data flow, communication protocols, handovers, etc., is proposed. Physical phenomena, such as heat propagation, cannot either be represented using the appropriate dynamical model. Network and physical simulators, on the other hand, present the opposite problem. The inclusion of all these details into a unique simulation causes simulators to be heavier and slower, but results closer to reality are obtained.

In that way, as previously mentioned, works about methodologies for designing co-simulation tools are scarce. In fact, most works are focused on the implementation process, which it is a later stage to the design phase. Thus, most existing ones are focused on very particular aspects, such as the coordination of continuous and discrete-events simulators [61], the problem of mixing tools implementing time with different data structures (and accuracy) [62], or the creation of a multi-domain description language adequate for the co-simulation [63]. Works on requirements capture can be also found [64,65] and final users’ needs evaluation [66,67], but they are focused on generic software, often resulting inefficient. Finally, papers addressing a complete methodology focused on the design process usually investigate procedures to create equivalent models in order to load the entire scenario (including all domains) in a unique simulator [68] (for example, representing people as mobile nodes in the network simulator). We are sure that no work proposes a complete methodology which allows software architects to design their own co-simulators.

In the next subsection, we review the state of the art on the simulation of the three main domains which compose a CPSS.

### 2.4. Social, Physical and Network (Cyber) Simulation

Although systems including five different domains in CPSS have been reported [1], typically they only include three subsystems: physical world, cyber world and social world [2]. Thus, in the context of CPSS simulation, basically three types of proposals should be taken into account: the physical processes simulators, the social simulators and the IT simulators.

The **physical processes simulators** are based on mathematical engines which calculate numerically the evolution of the system integrating the differential equations which describe the system’s behavior [63]. These simulators are the oldest [69,70] as they were extremely used in manufacturing, avionics or metal factories. Nowadays, it is difficult to find research works on this topic, as a great variety of powerful commercial products may be found. Simulink [71], MapleSim [72], OpenModelica [73], Wolfram SystemModeler [74] or xcos [75] are some of the most famous and used examples. All these solutions provide a graphic interface and a programming environment which recovers the mathematical details and enable high-level developers work comfortably.

In this area, current research works try to include some aspects of the virtual world in the physical simulations. These solutions are known as mixed simulation [76]. However, most of these proposals are focused on sanitary scenarios and are difficultly extended to other applications, as specific knowledge and configurations is required.

On the other hand, first CPS (Cyber-Physical Systems [77]) simulators have been proposed [78,79,80]. In the final design, these simulators will include the physical and the cyber world; however, nowadays they only consider the physical world and some characteristics about the cyber world. Furthermore, they cannot recover the mathematical details, so experience in numerical integration is required in order to correct manage the simulations.

In both cases, mixed and CPS simulations, the techniques used in order to include the cyber world in the physical one consist of deep modifications in the numerical algorithms and models. Moreover, in general, physical processes simulators integrate the dynamical models for total amount of simulated time and, once obtained the results (consisting of one or various discrete signals); they are showed in the graphic environment or returned in the method callback. Table 2 compares all the simulators described in respect with the most important characteristics. It is important to note that other types of simulator may be found. For example, very recently various cyber physical systems modeling frameworks aiming at modeling different physiological processes have been proposed [81,82]. Moreover, prior works in Cyber Physical systems which aimed at modeling stochastic variables with long tails which are more adequate to model human behavior have also appear [23].

**Social simulation** studies the interaction among social entities taking into account their psychology and their behavior, their interactions with other entities in the environment and the behavior of all entities as a group [83]. Mainly, there are two different types of social simulations. First, social-level simulations analyze situations, usually a society, as a whole, including its evolution when a change happens. Second, agent-based simulations study each person in a more detailed manner, designing a specific model for each agent (for example, the movement becomes an important factor in the simulation). In CPSS environments, agent-based simulations are preferable, as may include factors such as the movement of the individuals. On the contrary, social-level simulations are not suitable at all as important characteristics of people (movement, etc.) cannot be considered.

In agent-based simulations every element in the simulation is modeled as an agent, a unit that acts on their own, without external direction, in response to situations they encounter during the simulation [84]. An agent is updated every certain time according to the behavior defined inside its programming, what usually means some movement of the agent and maybe the influence and interaction with some other agent. Some of the agents can be very primitives and actually have nearly no behavior (such as a table in the middle of the room), while others (such as people) can be very complex.

In general, agent-based simulators deal with a high number of autonomous people, who enter, move around and leave a large installation, interacting with each other and with the environment (which consists of many devices with communication capabilities). Features related to autonomy, interaction, mobility [85] and openness can be achieved by employing agreement technologies [86], as well as semantic alignment [87], negotiation [88], argumentation, virtual organizations [89] and trust and reputation mechanisms [90].

Various kinds of agent-based social simulators may be found. Some of them simulate human intervention to react to events produced by sensors in the scope of Ambient Intelligence. This is the case of Ubiwise [91], TATUS [92], UbiREAL [93], and OpenSim [94]. In this sense, the DAI Virtual Lab [88] is complemented with a living lab which allows researchers to continue their work in a physical environment. These frameworks cope with the modeling of realistic environments and not only the wireless sensor networks.

Other social simulation environments can assist in the development of IoT systems by simulating artificial societies of users immersed into the intelligent environment and generating sensors events [95]. Although social simulation has been used for testing a number of closed systems, mainly in the emergency management scope, the general use of this technology for providing general AmI systems with automatic testing is still a novel field with few contributions such as UbikSim [96] and Phat [97] simulators.

Traditional works on social modeling and simulation try to obtain an analytical model to represent a certain human process [98] (such as the traffic flow [99] or crowds of pedestrians [100]).

In the last years, research works about agent-based social simulation have focused on discovering or predicting new models and behaviors, instead of on designing new simulators. Actually, there exists various professional generic agent based social simulators (apart from those which have been designed and particularized for a certain case study or application): MASON, Repast [101] and Swarm. Besides, most of these instruments provide a graphic environment, some of them based on 2D representations while others have a 3D tool [102]. Usually, in a simulator, users can run a predefined scenario by modifying some of the parameters and creating approximately the same predefined agents to see the differences between the simulations. This use of the tool requires no programming ability because the simulation can be controlled with a GUI that firstly enables the user to modify the parameters and specify how many agents are going to be simulated and, once the simulation starts, it enables pause, stop and resume the simulation while it is running. These simulators also enable some kind of batch simulation [103] where you can run some hundreds of simulations with the same or different parameters to later analyze the results generated in a log.

In order to create a different scenario and different agent models some programming is required to define the agent behavior and place the different elements in the environment. In this case, specific knowledge of the language and the behavior models are required in order to create the desired simulation despite you can create a behavior composed of several predefined behaviors

Table 3 compares all the social simulators described above.

Finally, **cyber world simulators** (known as IT simulators or networks simulators) include, probably, the widest catalogue of simulation solutions among all the CPSS subsystems. On the one hand, it may be found the traditional networks simulators, such as the NS3 [104]. These simulators must be configured using a generic programming environment, as they usually do not present a graphic interface. Sometimes, it is possible to show the result graphically using third-party tools. These simulators are developed professionally, so research works are not usually focused on them. There are several simulators nowadays both open-source such as NS3 or OMNet++ [105] and proprietary such as OPNET [106] or NETSIM [107].

On the other hand, new instruments for the most recent and popular concepts on IT sciences, such as the Internet-of-things (IoT), have appeared and could be used in CPSS simulations. These proposals are still been developed and many researchers are working on this topic. Some examples are the IoT simulator Cooja [108], SimpleIoTSimulator [109] or AutoSim [110]. These tools used to incorporate a graphic environment to create the scenarios. However, the customizing options are much fewer than in traditional networks simulators. Social IoT simulators are a special case among the IoT simulators [111]. In these instruments, some aspects of the social human behavior are included in the models in order to, for example, represent the human movements.

All IT simulators are event driven, “jumping” from one event to the following event in the simulation process. During the simulation, the logs generated from the processing of each event are presented (or stored) at each simulation step.

Table 4 compares all the IT simulators described above.

## 3. Methodology for the Implementation of Application-Specific Cyber-Physical Social Sensing Co-Simulators

This work proposes the first methodology for the design of CPSS co-simulators. The proposed methodology supports the work of software architects, which is the first approach to the co-simulator creation. At this stage, generic discussions about the complexity of simulations to be performed are proposed; general questions about requirements, the users’ profile, and the development team must be answered; and the global lines of the future implementation are stablished. In later phases (at implementation time) more specific problems (which are not the focus of this work) should be addressed. In particular, crucial aspects about the data formats and time representation should be solved.

In order to organize these activities, we propose a four-step methodology for designing a CPSS co-simulator being able of fulfilling the users’ needs and requirements:

1. Selection of the co-simulation paradigm
2. Particularization of the general simulation model and simulation lifecycle
3. Selection of the appropriate coordination mechanism
4. Design of the user interface and results presentation

Several authors have reported the interest of research on co-simulation. The possibility of simulating individual components using different simulation tools simultaneously and collaboratively enables stakeholders to evaluate deployments in a very realistic way. In order to reach this objective, individual simulation tools should be able to exchange information such as variables and their values, synchronize the time steps and, in general, orchestrate the co-operative simulation [112]. Mainly, two standard technologies have been proposed to coordinate co-simulations: the High-level architecture (HLA) [56] and the Functional Mock-up Interface (FMI) [57]. Although several tools are compatible with these standards, not all simulation tools implement the same and, even, some tools do not implement any (for example, most social simulators). Moreover, problems and restrictions in the integrated tools based on these standards have been reported [112]. And finally, some important challenges related to CPSS co-simulation are not addressed by these solutions (such as data exchange and time management in the case of FMI standard). In fact, the complexity of CPSS pushes designers to create application-specific and simple solutions, losing most of powerful advantages of standard solutions.

In that way, the proposed methodology in this paper aims to enable CPSS researchers and software architects to design the most adequate co-simulation for a given application, addressing the four most important challenges. Namely [112,113]:

- The first step addresses the “Data Flow and Concurrency”. Different paradigms in order to parallelize and manage the data flow among the domain specific simulation tools are presented and different criteria to select among them depending on the situation are provided.
- Challenges related to the “Modeling language” and “System Topology representation” are addressed during the second step. A basic simulation model is proposed, and indications to adapt it to the particular scenario under study are provided. An identical process is followed with the simulation lifecycle.
- “Time management” and “System Scalability” are investigated in the third step. Different options to coordinate the different time representation and simulation speeds are studied. Besides, depending on the desired future system scalability and the scenario under study, different criteria to select the most appropriate management technique are provided.
- Finally, the fourth step is focused on “Tool heterogeneity”. In order to homogenize the interaction with the proposed co-simulator as much as possible, different ideas about the possibilities for user interfaces are provided and analyzed.

**Figure 2:**
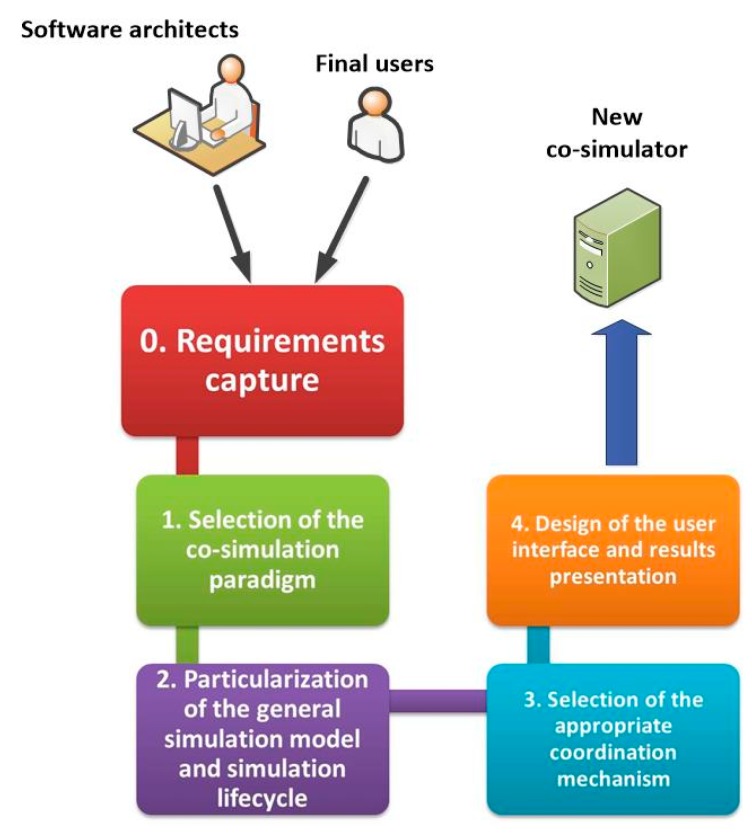
Scheme of the proposed methodology.

As a previous phase to the application of the proposed methodology, an interaction process among software architects and final users must be performed. Our proposal also includes and regulates this process and helps both, final users and designers, to determine the requirements and characteristic applicable to the new co-simulation tool (see Figure 2). The implementation process, although it is the most time consuming task, is a post-design activity, so it is not considered in this work.

In this section, the four steps of the proposal are explained, and the previous phase of requirements are captured to obtain from final users the characteristics which the new co-simulator should meet.

In relation to traditional software engineering methodologies, the proposed technique lacks an explicit mention to a software evaluation step. However, this phase should be considered. Once the new co-simulator is designed, its functionalities should be evaluated to prove it fulfills the user requirements. If not, a new iteration following the proposed methodology should be carried out, considering the new user inputs.

### 3.1. Previous Phase: Final Users’ Requirements and Characteristics Capture

Many works have studied the requirements that co-simulation tools should fulfill in order to operate correctly and efficiently [25,30]. However, in application-specific tools, most of these generic requirements could be inapplicable and other needed characteristics may not be considered. Moreover, the interaction with final users may be complicated and very time consuming if an ordered process is not followed. Furthermore, not only traditional requirements (such as the tool’s accuracy) should be considered. In particular, characteristics of the development team, final users’ education and the type of simulations to be performed should be defined.

In this previous phase, software designers and final users should meet and stablish the requirements the new simulation tool should fulfill, and other important characteristics. Four important thematic blocks must be addressed: technical requirements, application requirements and characteristics, development team characteristics and final user characteristics.

In respect to technical requirements, all of them may be associated to one property among the following four [25]:

- Flexibility. The adaptation level of the proposed solution to new usages, utilization modes, technological instruments, etc. must be determined. For example, if required, the simulator should be able to be applied to new scenarios.
- Modularity. Depending on the expected use for the co-simulator, the tool should include several modules independently handled. For example, if applying many changes in the simulator structure during its operation is desirable, modules and components should be easily added and removed without affecting any other part of the tool (total modularity).
- Scalability. The upper limit for the simulation scenario complexity should be defined. For example, the maximum admissible number of agents in a particular scenario, or the level of complexity of the agent’s behavior should be determined.
- Accuracy. Users should be able to select the desired accuracy level. For example, users must indicate the maximum time without updating the simulation (which allows calculating the required time step at the implementation stage, see Section 3.4)

Requirements about flexibility and modularity refer to the programming structure of the co-simulator. In particular, they indicate the implemented level of openness with the co-simulator. Scalability implies that the resulting co-simulator should be able to simulate future scenarios. Finally, accuracy enables users to configure the simulations according to particular configuration parameters.

In respect to application requirements, they also match the previously presented properties. Finally, characteristics related to the application scenario, the development team and final users should be considered. Relevant information about these topics should be discussed among designers and final users, in order to adapt the new simulation tool to the specific context. Table 5 presents some basic characteristics which should be considered, although others could be added.

The first and third steps allow generating co-simulators which fulfil flexibility requirements. Co-simulation paradigms and coordination mechanisms are open solutions which allow easily modifying the programming of the co-simulator. Second step is designed to meet modularity and scalability requirements. The simulation model and the simulation lifecycle can be modified, extended or reduced in an independent way, and according to the users’ needs. Finally, accuracy requirements are mainly supported by the fourth step. An appropriate graphic interface must enable users to configure the simulations. Relevant characteristic may affect all steps in the methodology.

The next subsections describe deeply the four main steps of the proposed methodology.

### 3.2. Selection of the Co-Simulation Paradigm

In the first step, once software designers have selected the domains involved in the simulation, requirements and characteristics have been captured and the available tools which are going to be used to compose the final co-simulator, it is necessary to select the co-simulation paradigm on which the co-simulator is being based. An explanation about all the possibilities, and the criteria for selecting the most adequate, are provided in this sub-section. It is important to note that most of these criteria are extracted from requirements captured in the previous phase (see Table 5).

**Figure 3:**
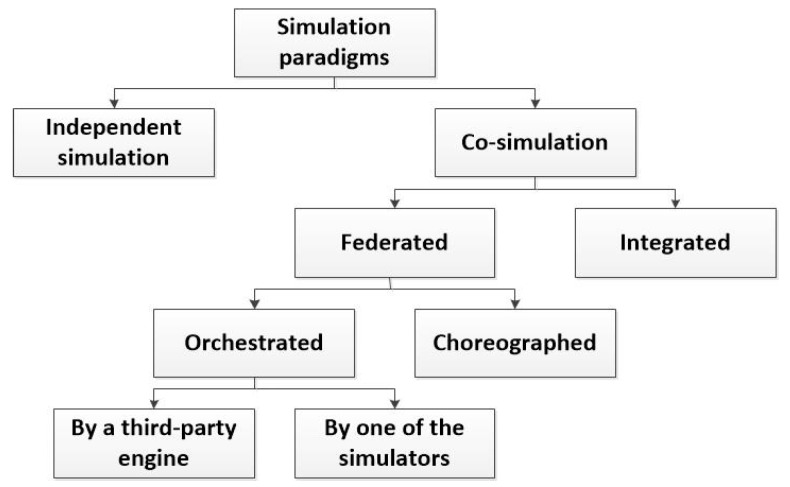
Taxonomy of CPSS simulation paradigms.

Figure 3 proposes a taxonomy of the simulation paradigms, which could be applicable to the CPSS simulation.

**Figure 4:**
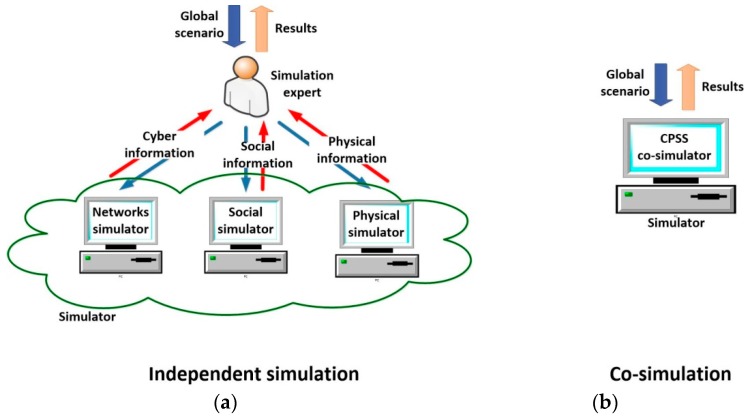
Different simulation paradigms (**a**) Independent simulation (**b**) Co-simulation.

In general, two different types of simulation scheme may be implemented, when various domains compose a global scenario: *independent simulation* or *co-simulation*. In the *independent simulation* each domain is simulated in a different domain-specific simulator. In this case, once designed the global scenario, an expert must divide it into the different subsystems (three, in the case of CPSS—the physical, the cyber and the social worlds-) and configure each simulator separately (see Figure 4a). Finally, the results obtained from each tool must be combined with the others in order to generate the global results for the proposed scenario. As main advantage (see Section 3.4), if various work stations are available, it is possible to run all the simulations in parallel (which is very efficient in time).

On the other hand, *co-simulation* tools are instruments being able to process a description including all the characteristics of the global scenario (see Figure 4b). They may be implemented using different integration levels, which, at the end, causes the simulation time goes up in different quantities depending on the implementation selected. In any case, considering the same hardware platform, these solutions always require a higher simulation time.

As we said in Section 2, recent attempts to reach a co-simulation tool for two of the CPSS subsystems are based on the deep integration of new characteristics in the existing simulator. We call this co-simulation paradigm *integrated co-simulation*. Integrated co-simulation tools are monolithic instruments where none separated functional part can be distinguished. In practice, they can be understood as a unique simulation algorithm. In general, this is the final objective when building co-simulation tools as, at that moment, the resulting solution is completely different from the parts. However, it is really difficult to create an integrated simulator right from the scratch. In fact, various previous works [113] have showed the complexity of combining various tools and domains in a unique simulation (even if only two different tools are considered).

In contrast, we define the *federated co-simulation*. In *federated co-simulation* various (three in CPSS—a social simulator, a network simulator and a physical process simulator-) domain-specific tools are coordinated to simulate the global scenario. Each part could be modified in different levels, but there is always a separation among the different functional parts (which maintain all their traditional characteristics). Federated co-simulation is the most profitable paradigm in the first stages for building a new simulation tool.

Now, as we said, in federated co-simulation the different parts which make up the global simulator could be modified in different level. Depending on the number of required modifications different federated co-simulation paradigm may be identified.

**Figure 5:**
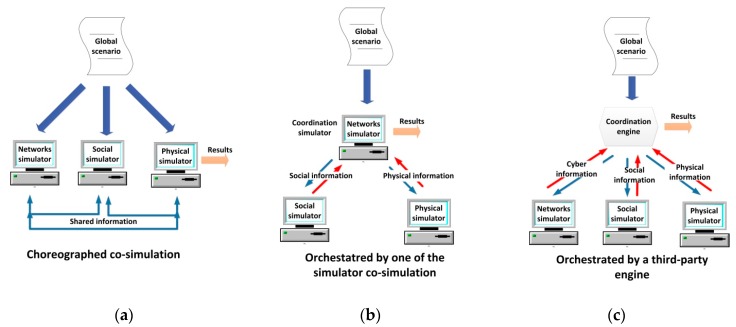
Different simulation paradigms (**a**) Choreographed (**b**) Orchestrated by one of the simulators (**c**) Orchestrated by a third-party engine.

The paradigm which needs the highest level of development is the *choreographed federated co-simulation.* In this scheme, all the different domain-specific simulators have been modified to understand the global description of the simulation scenario, to select the important data for them, to configure the simulation and to share the resulting data with the other domain-specific simulator (see Figure 5a). Thus, programming is required in order to modify the simulation tools. Finally, one of the simulators is in charge of building the final simulation results. This federated paradigm is the closest to the integrated simulations.

In contrast to choreographed co-simulation, *orchestrated federated co-simulation* does not require to modify all the domain-specific tools (or the modifications are negligible), and, then, programming knowledge to modify the simulators themselves is not required in the development team. In this paradigm, an entity is in charge of receiving the global description of the scenario, processing it, and configuring the simulation in the different tools. Finally, this entity must also generate the final results using the data from the different simulators. In *orchestrated by one of the simulators federated co-simulation* the described entity is one on the domain-specific simulator which has been adequately modified (see Figure 5b). In *orchestrated by a third-party engine federated co-simulation* no modification in the domain-specific tools is required, as the described entity is a third-party program which uses the available interfaces in the simulator to interact with them (see Figure 5c).

Various proposals and commercial simulators applying different paradigms may be found. For example, in [114] it is described an orchestrated by a third-party engine federated co-simulator for CPS (based on the union of the NS3 simulator and the MASON simulator). In [115] an orchestrated by one of the simulators federated co-simulator is presented. The proposal is based on the coordination of MATLAB/Simulink (a tool which, besides, performs the coordination tasks) and the NS3 simulator. This paradigm is greatly extended (especially when MATLAB/Simulink suite is involved), for example to perform simulations about reflector antennas with the commercial software GRASP [116]. On the other hand, in [117] a Smart grid simulator based on choreographed co-simulation between the NS2 simulator and the PSLF (Positive Sequence Load Flow, software for power systems) Simulator is described; and in [118] an integrated simulator (also for Smart grids) is presented.

Selecting the appropriate co-simulation paradigm for a new simulation tools is a complicated task which depends, basically, on two factors: the technological capacity of the future developers and the future users’ abilities and (mainly) the characteristics of the selected tools to make up the co-simulator. Table 6 presents and discusses the most important criteria which must be considered in the selection of a co-simulation paradigm.

### 3.3. Particularization of the General Simulation Model and Simulation Lifecycle

Once selected the most appropriate co-simulation paradigm, the application for which the simulator is being designed must be considered. In that process, characteristics and final users’ requirements captured in the previous phase (see Section 3.1) should considered as inputs. In particular, the application scenarios and the type of simulations the users are going to perform should be taking into account. The first element (application scenarios) will allow designers to describe a specific simulation model. The second one (the type of simulations) might help to propose the simulation lifecycle most adequate. The second step in the methodology is dedicated to these two tasks.

#### 3.3.1. CPSS Simulation Model

The simulation model on the proposed designed co-simulator must be focused on the most relevant aspect of the reality for the particular selected application. Elements from all the subsystems may be present, but description should be much more exhaustive when representing certain parts of the world.

**Figure 6:**
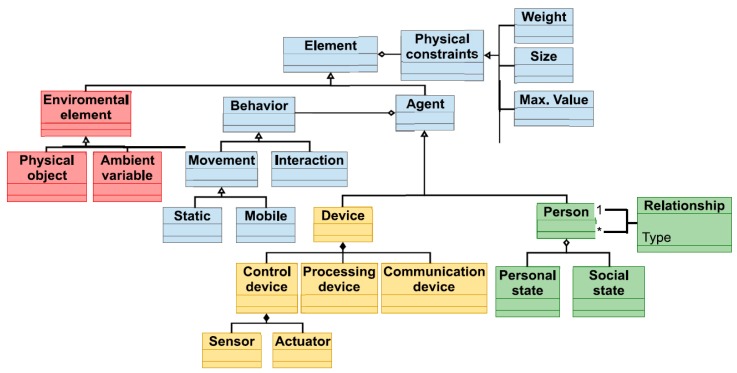
Base CPSS simulation model.

Figure 6 represents a basic simulation model for CPSS which can be taken as initial step in the construction of an own model. In the presented basic model four types of elements are present: in blue, general elements relating the three worlds (physic, social and cyber), in red elements from the physical world, in yellow devices belonging to the cyber world and, in green, elements in the social world.

As can be seen, general elements include the constraints and the behavior of every agent in the simulation. Social elements basically consider people, their state (particular and as a group) and the relations among them. Cyber elements refer a list of devices including sensors and actuators. The rest of agents present in the scenario belong to the physical world.

Using the basic model in Figure 6, the most adequate simulation model for the specific application under work must be developed. For that, some parts of the model may be reduced (for example, distinguishing between sensors and actuator could be unnecessary), and others extended (including, for example, more elements for composing a person).

Some methodologies for the creation of correct and complete simulation models have been proposed [119,120]. Basically, depending on the application and purpose of the designed co-simulator some aspect of reality could be abstracted away [121]. However, in Table 7, some general lines are given in order to build the simulation model according to the selected application, the scenarios under study and the specific problem being addressed with the co-simulator. For unreported applications nowadays, actions to be taken would be pretty similar. Thus, as main contribution to the agent-based modeling we propose a guide for software architects (as well as an initial model mixing, for first time, the three basic CPSS subsystems), in order to allow them to create the most adequate model in each case (considering current and future scenarios).

#### 3.3.2. Simulation Lifecycle

Depending on the type of the simulations being performed in the designed co-simulator, the simulation lifecycle may vary (more steps could be added, for example). The steps included in the simulation lifecycle are going to affect, overall, the fourth step in the methodology, dedicated to the interaction with users. For example, if all the simulations to be performed only address one goal, no step for “goal specification” is needed, and no interface dedicated to this step is required.

In general, designing the most adequate lifecycle is much easier (almost elemental) than building the simulation model. Next, a complete generic simulation lifecycle is described, from which different steps can be removed (if necessary), or where new states may be defined.

The creation of the particularized simulation lifecycle is very simple: if the tasks associated with a certain state described below do not have to be executed, this state is removed; if new tasks have to be added, new states should be defined.

**Figure 7:**
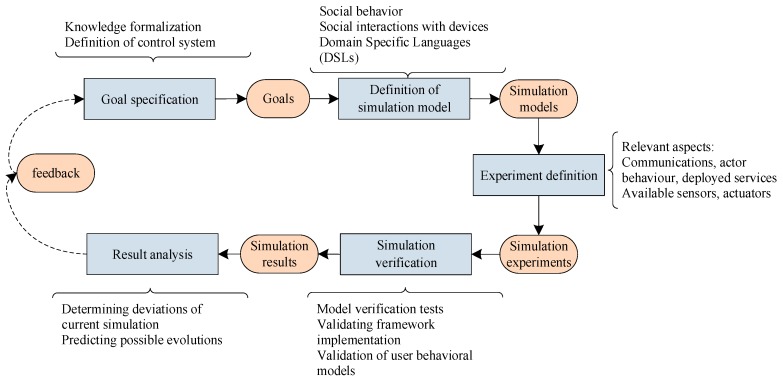
Basic simulation lifecycle.

Co-simulation environments are often used to test how humans relate to devices and other infrastructure objects. Simulations showing specific aspects of these relations are executed and redefined multiple times to retrieve knowledge about users and devices. The process is represented in Figure 7 and the quality assessment [128] of every step is described below.

**Goal specification**: Stakeholders meet to determine the specific problem to solve, capturing this information by field studies and/or other requirement acquisition techniques. The research problem is formally and clearly formulated. One goal commonly pursued in simulations integrating social behavior and device interaction is to check the functioning of a control system (a device or set of devices that manages and regulates the behavior of other systems in the smart environment), analyzing if this system correctly takes into account its execution context. This is a prior step to define the simulation model associated to this system, as analyzed goals can be used to model what kinds of experiments are desirable for the particular problem and the type of situations considered.

**Definition of simulation model**: Social behavior and social interactions with devices and the control system must be integrated into the simulation model. Some considered models (see Figure 6) are *person*, *behavior* (describe individual and collective human behavior), *environmental elements* or *actuators*, *sensors* (describe the physical characteristics of the AmI environment).

Domain Specific Languages (DSLs) are used to create simulation models by model-driven development techniques. Other end-user development tools present in the co-simulation framework will be used to transform these models to simulation specifications. These tools are reusable across domains and permit the developer or any other stakeholder to describe specific aspects of the problem.

Some questions arise regarding the correct definition of the simulation model. Are there many simulation models in the literature or this is the very first of its kind? Is the underlying social theory correctly instantiated using proper methods and algorithms?

**Experiment definition:** Simulation experiments take into account the selected models depending on the aspects to be addressed. It is foreseen to take into account the agent behavior, and all the key elements included in the particular developed simulation model. Simulations addressing one or more of these aspects will be produced and evaluated by the developers. The required simulations will be described by the experiments model and will incorporate specific information, such as building blueprints or device limitations.

Experiment definition is done via configuration tools. Particular applications are chosen and different models are selected (users, populations, spaces, actuators, sensors), and combined, and then the different experiments that are going to be applied are prepared. Experts would facilitate end-users the task of creating those experiments by reusing existing information and allowing them to decide which information should be extracted from the simulation, for its later analysis. The result of applying these tools would be simulation specifications.

Simulation execution in co-simulation system: The problem to be simulated is parameterized, producing the problem specific components the reusable co-simulation framework requires. Actions from the experiment definition and control system behavior are applied on the simulation so as to affect the simulated actors and to product the expected effect. The system would be adjusted to deal with the different situations that might occur: necessity of repeating stimulus (without saturating the audience), unexpected problems with devices, etc. Besides, communication among devices is tested, assessing effectiveness and cost. Possible problems like bandwidth, transmission quality, etc., must be also validated.

**Simulation verification:** A verification step is required to ensure that simulation model and experiments were well-defined. In this step passive and active tests are conducted to verify that the model is behaving in the way it is intended to behave (internal validity). Also, some tools and techniques such as code debugging, unit testing, profiling [129] as well as test suites (histograms, spatial analysis, pattern matching, etc.) must be considered, as these techniques allow us to validate the proposed model and the implementation of the framework. Some questions arise: In terms of the definition and implementation of social behavior, is the underlying social theory correctly instantiated using proper tools or programing languages? In terms of computational efficiency, how efficient is the implementation of social behavior in terms of using computational resources? Is there any architectural implication in the publication and acquisition of user behavioral models?

**Results analysis**: Experiments are analyzed according domain knowledge to improve our current model and identify new needs. When the experiments are successful, an exhaustive documentation is thus available about how the system is wanted to behave. The work carried out can be used to track the behavior in the real setting.

Regarding the deployment process of services or control systems in the CPSS environment we assume that the system tested against simulations would be the same as those installed in the real environment. Using a software-in-the-loop approach, we would ensure that the conditions of these services in the simulation are the same or sufficiently similar to reality.

Once deployed, information collected during experiments can be used to determine whether current observation in the real AmI environment corresponds to a simulated scenario or whether it is necessary to launch a simulation with the current context in the real world so as to determine deviations with regards to experimentation and/or predict possible evolutions from the current situation.

Some questions arise: In terms of overall effectiveness, does the model render what is necessary for answering the initial research questions? What is the quality of the simulated infrastructure that renders the most effective simulation experience?

Finally, in order to correctly follow the defined simulation lifecycle, we need to take into account the following requirements:

- In CPSS, we need to consider several aspects (e.g., social, physical and cyber) of the agents’ behavior. For example, movement in devices can be dictated by movement of people carrying those devices. Also, in a social simulation it is useful to know if persons that walk through a corridor should have enough WiFi coverage, or would detect a Bluetooth beacon that is broadcasting a signal in one of the surrounding rooms.
- Some tools must be needed to create experiments from previous ones, by modifying functionalities or stimulus affecting simulated actors to produce expected effect. Experiment creation should be done by domain experts, who have the required experience to identify human and device behavior in knowledge bases but they also have little experience in software engineering. Thus, it is needed to provide configuration and personalization tools easy to use for domain experts and adequate to their skills.
- Tools for analyzing the results of the simulation are extremely important to deal with the amount of data that is produced by the simulation. They should be able to process and facilitate the analysis by the experts. Moreover, after real deployment of services or control systems, those tools should also be used for analyzing and comparing current data against simulations so as to identify deviations and foresee future situations. For doing that, performance data must be generated by the simulator.

### 3.4. Selection of the Appropriate Coordination Mechanism

Some of the proposed co-simulation paradigms do not require any additional decision, as the paradigm determines the entire structure of the new co-simulator. In generic designs, issues such as the data format or the communication protocols to be employed in the coordination process must be selected. If a choreographed paradigm is selected all simulators involved must be totally compatible, so no action about the data format or protocols is required (all of them should implement the same). Again, orchestrated co-simulation requires the orchestrator to implement all the protocols, interfaces, etc. employed by the rest of tools, so no action about the data format or protocols is required as well. In federated co-simulation, the key topic is the coordination of the different domain-specific tools which compose the global co-simulator. Thus, only decisions about the mechanism to synchronize the execution of the domain-specific simulators must be taken. The third step of the proposed methodology is focused on this point. In particular, two different mechanisms could be implemented (see Table 8).

**Figure 8:**
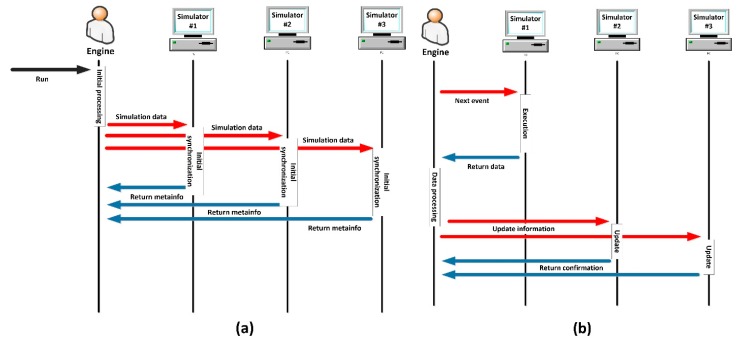
(**a**) Sequence diagram describing the initial synchronization process in a co-simulator; (**b**) Example of a sequence diagram about the stop-and-wait coordination mechanism when using a federated co-simulation controlled by a third-party engine.

The coordination mechanism is, probably, the most critical step in the proposed methodology. Although the design process can be simple (see Table 8), at implementation time, programming these policies forces to face various crucial problems. For instance, the event ordination if various discrete time domain-specific simulators are employed, or the definition of a common time representation when each tool employs a different data format (and accuracy). The selected solutions for these challenges depend on the chosen co-simulation paradigm at the first step, but (in general) an initial synchronization process must be performed (and configured) in order to homogenize time characteristics and variables (see Figure 8a). Then, simulation may be executed normally—evaluating the system state after each event or time step—(Figure 8b).

In order to select the most adequate coordination mechanism two factors should be considered: the difficulty of implementing the mechanism and the time required to perform a simulation when using a certain mechanism. This second criterion is directly related with the scalability requirements acquired from final users in the initial phase (Section 3.1). In general, implementing a parallel execution is much costlier than a stop-and-wait model. However, the time required to finish a certain simulation in smaller if parallel execution is considered. In order to evaluate these temporal factors, a theoretical analysis is provided below. In particular, Equation (9) provides a theoretical analysis in order to optimally select the best approach for specific situations (depending on the expected number of simulations to be performed, etc.)

We are supposing a simulation about a global scenario $S_{G}$ represented by means of a global model $M_{G}$ composed of information about three different domains: the physical, social and cyber world (1).

(1) $$M_{G}=\;\left\{m_{ph},m_{so},m_{cy}\right\}$$

We are calling $T_{ph}$ to the needed time to simulate the physical model $m_{ph}$ in a physical processes simulator; $T_{so}$ to the needed time to simulate the social model $m_{so}$ in a social simulator; and $T_{cy}$ to the needed time to simulate the cyber model $m_{cy}$ in a networks simulator. All these variables, $T_{ph}$, $T_{so}$ and $T_{cy}$, represent stochastic processes which may be understood as ergodic processes whose statistical mean is referred as $E\left[T_{ph}\right]=\;\mu_{ph}$, $E\left[T_{so}\;\right]=\;\mu_{so}$ and $E\left[T_{cy}\;\right]=\;\mu_{cy}$. Calculation time is a stochastic process as aleatory facts (such as numerical noise) may affect the calculation speed, the time step (if it is considered as variable), etc.

As reference, in average, the total amount of time required nowadays to simulate the global model $M_{G}$ using independent simulation $\mu_{T}^{indep}$ is (2) and (3).

(2) $$\mu_{T-s\&w}^{indep}=\;\mu_{ph}+\;\mu_{so}+\;\mu_{cy}+\;\mu_{pro\_scenario}+\;\mu_{pro\_results}$$

(3) $$\mu_{T-para}^{indep}=\;max\left\{\mu_{ph},\;\mu_{so},\;\mu_{cy}\right\}+\;\mu_{pro\_scenario}+\;\mu_{pro\_results}$$

In Equations (2) and (3), $\mu_{pro\_scenario}$ represents the mean of a stochastic process $T_{pro\_scenario}$ indicating the time an expert need to separate the global model in the different domains. In the same way, $\mu_{pro\_results}$ is the statistical mean of a stochastic process $T_{pro\_results}$ which indicates the required time to combine the obtained independent results. Both tasks could be performed automatically by using the adequate tools, but this implies a first initial version of a CPSS co-simulator for the application under study is available. For this work, no previous requirements are necessary, so tasks are considered to be performed manually. If the stop-and-wait coordination mechanism is implemented, the domain-specific simulations would have to be executed in a row and the total simulation time is indicated by (2). If parallel execution is available, the total simulation time is which indicated in (3). It is obvious that if parallel execution is available the mean required simulation time goes down, as $max\left\{\mu_{ph},\;\mu_{so},\;\mu_{cy}\right\}\ll \left(\mu_{ph}+\;\mu_{so}+\;\mu_{cy}\right),$ especially when the number of included devices and people goes up (as, typically, simulation algorithms present a complexity of order $\sigma \left(n^{2}\right)$). Another parameter which strongly affects the simulation time is the selected value for the minimum time step (usually simulation algorithms includes variable time step solvers). In most cases, the complexity order of the simulation algorithms depending on the time step is σ(n), so co-simulation tools usually present softer limitations related to the time step than which they present in respect to the number of considered devices. Moreover, the complexity of the simulation models and the attribute representation of agents may extend the simulation time, even in various magnitude orders (overall if non-linear models are included, which must be solved using complex numerical algorithms). In general, if more devices want to be included it is necessary to use simpler attribute representations and higher values for the time step. Obviously, as more complex models and smaller values for time step are employed, the precision in the obtained results go up. Now, in the case of an orchestrated federated co-simulation solution, a new stochastic process may be defined, representing the total simulation time $T_{T}^{orches}$ if stop-and-wait coordination mechanism is implemented (4). Moreover, another variable $T_{T-para}^{orches}$ may be defined (5) if parallel execution is available.

(4) $$T_{T-s\&w}^{orches}=\;T_{ph}+\;T_{so}+\;T_{cy}+\;T_{comm}+\;T_{init}+T_{end}$$

(5) $$T_{T-para}^{orches}=\;max\left\{T_{ph},\;T_{so},\;T_{cy}\right\}+\;T_{init}+T_{end}$$

In (4), $T_{comm}$ indicates the total time used in communicating the three domain-specific simulators (also a stochastic process, which includes, besides, delays due to feedback loops which are present in co-simulation tools but not on domain specific simulators). $T_{init}$ is the time used in configuring the simulation at the initial moment and $T_{end}$ the required time by the orchestrator for processing and presenting all the results by the simulators. In general, if mean values are considered, $\left(\mu_{init}+\mu_{end}\right)<\;\left(\mu_{pro\_scenario}+\;\mu_{pro\_results}\right)$, as the third-party engine may processes the initial configuration faster than any expert. Thus, in general, $\mu_{T}^{orches}<\;\mu_{T}^{indep}$.

The time expressed in (4) and (5) could be reduced if choreographed co-simulation solutions are considered. In that case, as no orchestrator is deployed, the time $T_{init}+T_{end}$ does not have to be considered. Then, the total simulation time in this case $T_{T}^{choreo}$ is represented by two new stochastic processes (6) or (7) depending on the selected coordination mechanism.

(6) $$T_{T-s\&w}^{choreo}=\;T_{ph}+\;T_{so}+\;T_{cy}+\;T_{comm}$$

(7) $$T_{T-para}^{choreo}=\;max\left\{T_{ph},\;T_{so},\;T_{cy}\right\}$$

Clearly, if mean values are considered, $\mu_{T}^{choreo}<\;\mu_{T}^{orches}\;$ in all cases.

Finally, as we said, in integrated solutions this step is not applicable as no coordination mechanism is needed.

As said, it is important to note that all previous temporal variables (in Equations (2)–(7)) are not fixed values. In general, the required time to perform a certain simulation is a stochastic variable which depends on many uncontrollable factors. Thus, relations among the variables may change in time. However, in order to support the decision making, in this section we are considering a typical case and the values can be fixed calculating the medium of various evaluations or by theoretical studies considering the algorithms complexity and the underlying hardware.

On the other hand, one additional temporal variable could be considered. Sometimes, the same event must be processed in various simulation tools, or events which are internal when using a unique domain-specific simulator must be externalize when this tool is included in a co-simulator. Then, the total time required to simulate each subsystem in a co-simulator (called, $T_{ph}^{c},\;T_{so}^{c},\;T_{cy}^{c}$) is, in mean, greater than the required time in independent tools ($T_{ph},\;T_{so},\;T_{cy}$). In particular, this situation may be reduced to the inclusion of and additional stochastic process $T_{co-sim}$ representing these activities (8).

(8) $$T_{ph}+\;T_{so}+\;T_{cy}<\;T_{ph}^{c}+\;T_{so}^{c}+\;T_{cy}^{c}=\;T_{co-sim}+\;T_{ph}+\;T_{so}+\;T_{cy}$$

The main disadvantage of this new variable is that it depends on the particular implementation employed in the new co-simulator. Thus, discussing its possible values is outside of the scope of this paper. Nevertheless, it can be seen that (although could be partial) the previous analysis is valid as the relations among the different variables are almost independent of the additional time $T_{co-sim}$.

For every co-simulation paradigm, as can be seen, the difference in the mean simulation time due to the implementation of a parallel execution or a stop-and-wait mechanism is, basically, the difference between $max\left\{\mu_{ph},\;\mu_{so},\;\mu_{cy}\right\}$ and $\mu_{ph}+\;\mu_{so}+\;\mu_{cy}$. Both values, in general, grows up with a complexity order of $\sigma \left(n^{2}\right)$ when increasing the number of agents in the scenario. However, the growth rate is higher for the expression $\mu_{ph}+\;\mu_{so}+\;\mu_{cy}$ as can be seen in Figure 8, where a generic graphic of the evolution of both expressions is provided.

**Figure 9:**
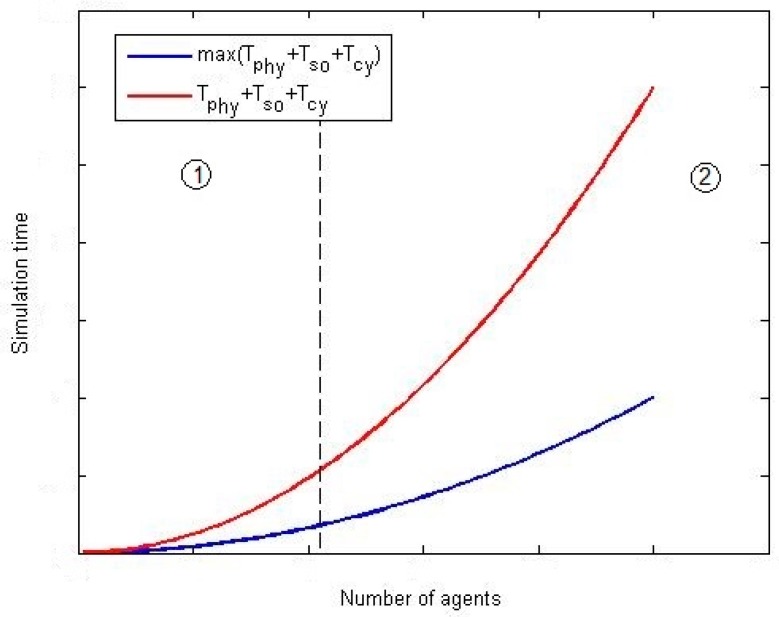
Generic graphic of the evolution of the simulation time.

In Figure 9 two areas may be distinguished. In the first zone, both expressions are low and not very different, as the difference between $max\left\{\mu_{ph},\;\mu_{so},\;\mu_{cy}\right\}$ and $\left(\mu_{ph}+\;\mu_{so}+\;\mu_{cy}\right)$ is not so remarkable. If simulations being performed are located in this area, stop-and-wait mechanism is preferable. Then, from a certain number of agents, the difference between $max\left\{\mu_{ph},\;\mu_{so},\;\mu_{cy}\right\}$ and $\left(\mu_{ph}+\;\mu_{so}+\;\mu_{cy}\right)$ starts growing and, in this area, parallel execution is desirable. In order to support scalability (in the limits of a certain application) it is very important to evaluate the area where the planned co-simulator will operate.

In order to evaluate the relation between the required simulation time and the development time to be invested, one new function can be defined (9). It represents the number of simulations which have to be performed in order to compensate the needed additional time to implement a parallel execution coordination mechanism.

(9) $$S\left(n\right)=\;\lceil \frac{T_{para}^{deve}-\;T_{s\&w}^{deve}}{T_{s\&w}^{sim\_n}-\;T_{para}^{sim\_n}}\rceil$$

In (8), n represents the number of agents in the simulation, $T_{s\&w}^{sim\_n}$ the required time to perform the simulation using a stop-and-waits mechanism, $T_{para}^{sim\_n}$ the required time to perform the simulation using parallel execution, $T_{para}^{deve}$ the needed time to develop a co-simulator using parallel execution and $T_{s\&w}^{deve}$ the needed time to develop a co-simulator using a stop-and-wait mechanism.

**Figure 10:**
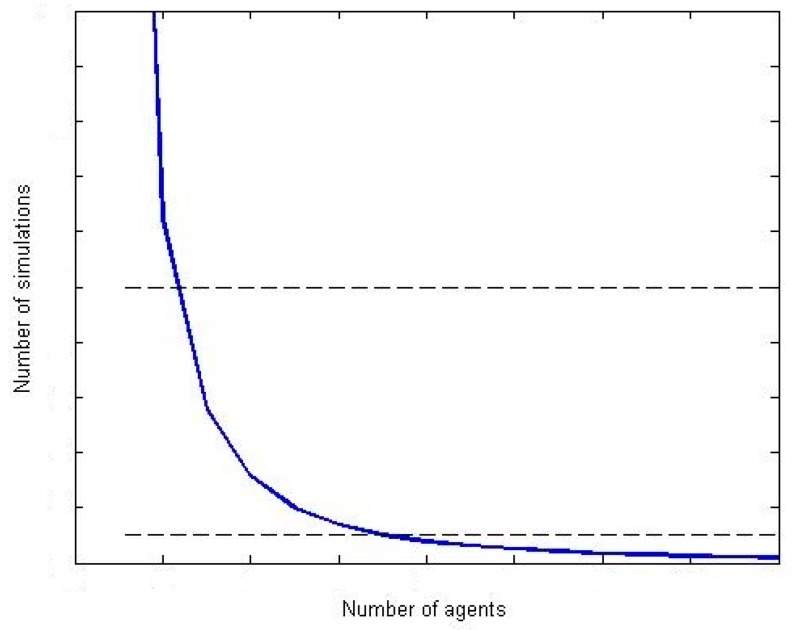
Function S(n).

As $T_{para}^{deve}-\;T_{s\&w}^{deve}$ is a fixed number and $T_{s\&w}^{sim\_n}-\;T_{para}^{sim\_n}\;$ presents a complexity order $\sigma \left(n^{2}\right)$, then S(n) presents a complexity order of $\sigma \left(\frac{1}{n^{2}}\right)$. Figure 10 represents this new function.

If simulations which are going to be performed include a great number of elements, very complex scenarios or devices extremely heterogeneous, parallel execution is the best option as S(n)<1. In this area, only one simulation requires an amount of time higher than which invested in implementing the parallel execution. On the contrary, if simulations to be performed are simple, the time invested in implementing a parallel solution does not make profit, as S(n)≫1. Finally, in the intermediate zone (when S(n)>1 but S(n) is not much higher than the unit) any of the proposed mechanisms can be selected to be implemented. In that point, the utilization degree may be determinant (e.g., if many simulations are being performed).

It is important to note that parallel execution does not imply to be able to parallelize various co-simulations. Domain-specific simulator may operate in parallel, but the co-simulator (as a unit) only performs one simulation each time. If parallelization capabilities are required, the adequate domain-specific simulation tools should be chosen (they must support parallel simulations) and, at implementation time, the coordination mechanism should also be able to manage various simulation at the same time.

Finally, more than the three basic subsystems of CPSS could be considered. Then, if more domain-specific simulation tools are included in the co-simulator, the expressions $max\left\{T_{ph},\;T_{so},\;T_{cy}\right\}$ and $\left(T_{ph}+\;T_{so}+\;T_{cy}\right)$ are generalized to $max\left\{T_{i},\;i\in simulators\right\}$ and $\sum_{i}T_{i}$. In this generalized expression, it can be seen that, when more domains are considered, parallel execution solutions are preferable (as the reduction in the simulation time, for any considered number of agents, is very remarkable).

### 3.5. Design of the User Interface and Results Presentation

The final step of our methodology focuses on designing the user interface customized to the particular application, simulation model, and lifecycle. Previous steps greatly influence this final phase. For example, if the “goal specification” state is removed from the simulation lifecycle it is not necessary to develop an interface to show that information. In conclusion, depending on the previous steps, the interface will adapt their elements to show only the relevant aspects of the desired simulation. Additionally, accuracy requirements established by final users in the initial phase should be also considered, in order to create the most adequate results presentation environment. Likewise, the final users’ profile should be also taken into account (see Table 5) to adequate the environment to their needs.

In every simulator, some universally required elements [130], must be always included in a user interface in order to perform a successful simulation (for example, there will be always an interface to start a simulation, stop it, or even pause it). Then, every interface for a simulator must include three tools: instruments to design a simulation, instruments to execute and control the simulation and tools to analyze the results. For each one of the three tools, some decisions must be taken (considering the most relevant aspects of the simulations to be performed with the co-simulator). We review these aspects below:

Instruments to design a simulation. User interface must permit the selection between all possible scenarios that can be found in the simulation process, this includes modeling and configuring all the elements that are present in a certain simulation. To allow this, a method to design the scenario has to be provided along with the necessary rules for a correct scheme. Basically, two options are possible: simulations are designed using predefined layouts or scenarios, or external instruments (such as programming environments) are linked to the co-simulator in order to create a new scenario with each simulation.

Instruments to execute and control the simulation. These elements are always available to interact, so the simulation process can be controlled externally. Depending on how the simulation has to be executed, the objective and if various simulations must be executed in a row, two types of tools can be used. Text interfaces and graphical interfaces are the primary options to be considered in the design of the execution and control interface. Any case, textual elements can be present in both types.

Tools to analyze the results. Results may be presented in a simulator in two manners. First there exists the “post-mortem” presentation. In this scheme, the simulation finishes creating a log file which is used later to construct and show the results (thus, graphical display needs a data source to represent the results). Secondly, in “live” presentation, results are calculated, processed and showed at the same time that the simulation advances.

Table 9 provides some criteria to select the most appropriate interface depending on the simulation that is going to be performed.

Finally, each co-simulator paradigm and even the particularization of the simulation model made on the first and second steps, require a specific set of tools to be able to configure and supervise the simulation. These elements can be added to the user interface in order to interact with the system involved in the simulation process. Some criteria to select the different items that a particular model’s needs are defined in Table 10, where we provide some key elements to add in each co-simulation paradigm.

## 4. Experimental Validation: Co-Simulator Development and Experiment Description

In this section, a practical validation for the proposed methodology is provided. We design (and implement) a particular CPSS co-simulator employing the proposed methodology, and, later, we design some experiments in order to evaluate its performance and the invested time its development; and compare those data with those obtained from other solutions. The correctness of the proposed solution will be deducted from the obtained results.

### 4.1. Co-Simulator Implementation

For this first practical use case of the proposed methodology, we decided to design a CPSS co-simulator focused on validating crowd management systems for emergencies (panic control, enhancing attention, etc.) in large facilities. These systems include the three main subsystems of CPSS: the physical world (buildings, stairs, exits, etc.), the cyber world (sensors, displays or speakers) and the social world (people, social behavior and other similar elements). General elements (see Section 3.3.1), such as time constraints, are present in all subsystems. These elements are transversal entities which condition the entire system’s operations and, then, they should be considered in all subsystems and domain specific simulations.

First, we perform the requirements and characteristics capture phase. In particular, requirements about the four properties described in Section 3.1 were stablished:

- REQ#1. Flexibility: The proposed simulation tool is focused in one particular application (crowd management), so requirements about flexibility are not imposed.
- REQ#2. Modularity: The proposed co-simulator should allow incorporating new types of devices in the cyber world as new technologies are proposed or investigated.
- REQ#3. Scalability: Simulations scenarios are limited to large-facilities so the maximum number of agents in a certain simulation is of various tens of thousands. As maximum, then, the co-simulator must be able to consider fifty thousands of agents. However, as we are saying later, that is not the most common case.
- REQ#4. Accuracy: As social models present a limited accuracy (human behavior is very difficult to predict), it is not required a high level of precision in the designed tool (a medium value would be acceptable).

Moreover, simulation tools for crowd management present some specific characteristics. Namely:

- CHAR#1. Simulations are performed by social experts, who are not programmers or technological professional. Thus, simulators cannot require technological knowledge.
- CHAR#2. In general, particular values or states at a certain time step are not interesting. In crowd management global tendencies (e.g., is the panic growing?) are more important than particular values.
- CHAR#3. The most important subsystem in crowd management systems is the social world. Simulations must provide precise social information, in order to evaluate the crowd behavior. Physical and cyber worlds are secondary.
- CHAR#4. As buildings may be complex structures, designing the models to include correctly the scenario in the simulator can be difficult in some occasions. However, although many agents might be included in one simulation, all of them present the same behavior, so the required processing capabilities to execute the simulations are limited.
- CHAR#5. The number of agents in a certain scenario is limited. Buildings are regulated and a maximum capacity is always defined. Even when over-capacity is considered, the number of agents in a certain scenario cannot increase indefinitely.
- CHAR#6. Results must be represented using both techniques: temporal and statistical graphics, and animations about the scenario’s evolution in time.

Apart from the previously presented characteristics, other circumstances must be also considered before implementing the co-simulator (final users and development team characteristics):

- CHAR#7. The group of future developers does not include any expert on simulators programming. Then, complicated and specific implementation cannot be addressed.
- CHAR#8. The simulation scenarios are limited to large facilities, so user don not have to be are not enabled to design their own scenarios.
- CHAR#9. In this case the employed domain-specific simulators were: Matlab/Simulink as physical processes simulator, NS3 as network simulator and MASON as social simulator. We chose these instruments due to their extended use in research, because they present an open architecture and, besides, MASON and NS3 are open source and, finally, due to their efficient performance.

Once the starting position has been exhaustively studied, it is possible to apply the proposed methodology in order to implement the desired co-simulator.

STEP #1. Selection of the co-simulation paradigm

First, as said in CHAR#7, no experts on simulators programming were available. Thus, integrated co-simulation got discarded (see Table 5).

**Figure 11:**
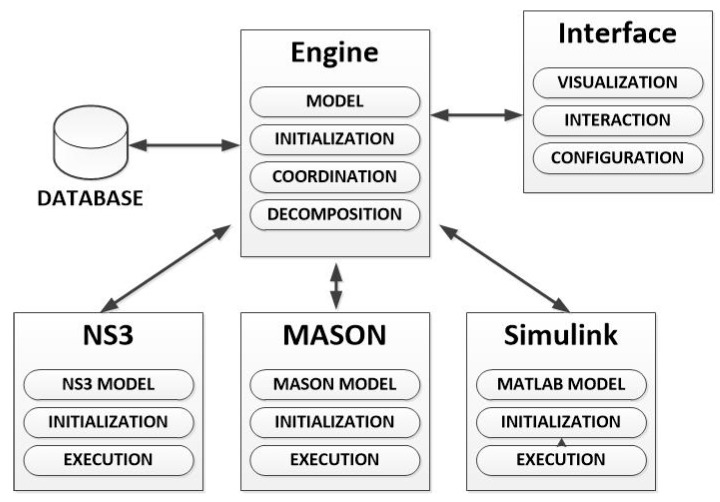
CPSS simulator architecture.

Besides, all considered simulators present an open architecture, so federated co-simulations are enabled. Secondly, in CHAR #9, it must be noted that MATLAB is not an open source tool, so choreographed co-simulation is not allowed. Finally, the results presentation and graphic interface provided with the selected tools do not meet the requirements of a crowd management system (in particular, any environment to visualize animations described in CHAR#6 is available). Moreover, CHAR#1 indicates that users are not programmers. Therefore, the proposed co-simulator must be implemented following the orchestrated by a third party engine federated co-simulation paradigm. Figure 11 presents the architecture of the proposed co-simulator. As a novelty, a database where all the logs are stored is also included (as we said, optional components could be added if necessary).

Additionally, NS3 simulator presents, by default, a modularity architecture, so REQ#2 is fulfilled, and any type of new devices could be easily added to the resulting co-simulator. Moreover, all the domain-specific independent simulators meet scalability requirement REQ#3.

STEP #2. Particularization of the general simulation model and simulation lifecycle

In this case, the simulation lifecycle is maintained as shown in Figure 6. No additional task is required and, as indicated in the characteristics cited above all the basic states are necessary.

**Figure 12:**
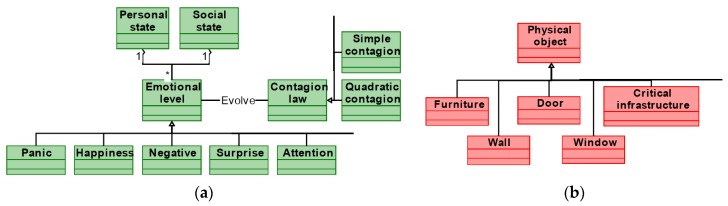
Extensions for the simulation model (**a**) Social world (**b**) Physical world.

In respect to the simulation model, CHAR#3 states that the most important subsystem in crowd management systems is the social world, so this area must be expanded in the model. Besides, as indicated in Table 6, the physical world should be also slightly complemented in order to represent in the proper way the scenario. Considering this both elements, Figure 12 shows the additional part to be included in the simulation model showed in Figure 6, by extending the relations of “Personal state”, “Social state” (both in green, as they are social elements) and “Physical object” (in red, as it belongs to the physical world).

In respect to devices (cyber world), which usually make up a very heterogeneous platform (the CPSS framework is known to have various types of device), the selected network simulator (NS3) has to be able to simulate all the required devices. In particular, see Figure 6, control, communication and processing devices have to be included. Different works [131] have proved that NS3 simulator may be used in that way, being possible to simulate from huge computing systems to small wireless sensor networks or cellular communications.

STEP #3. Selection of the appropriate coordination mechanism

In the third step, the coordination mechanism must be selected. In this case, it must be noted that three different simulation schemes are presented in the co-simulator: physical simulator makes a continuous simulation but the results are shared when the process finished; social simulator also makes a continuous simulation and shares the results each time step and, finally, network simulator makes an event-oriented simulation. However, at implementation stage, these three different schemes may be reduced to only two different schemes. In physical processes simulators, it is very common to recover the simulation routine using a program which runs the simulation each time step and shows the generated results. In that way, physical processes simulators (Matlab in our case) and social simulators (MSON in this works) behave in the same manner. Considering this situation, implement a parallel execution solution in the planned co-simulator would be a very difficult task.

On the other hand, as indicated in CHAR#4, the required processing capabilities to execute the simulations are limited. Moreover, as said in CHAR#5 the number of agents in the simulation scenarios is also limited. Thus, the simulation time tends to be small and the difference between $max\left\{T_{ph},\;T_{so},\;T_{cy}\right\}$ and $\left(T_{ph}+\;T_{so}+\;T_{cy}\right)$ is not so remarkable. We are waiting a high value for the function S(n). Therefore, stop-and-wait mechanism is preferable in our case. Next, the detailed design of the proposed coordination mechanism is explained.

When the engine receives the global model for the simulation scenario, it keeps track of the future events that are occurring in the different domain-specific simulators. The information about these events is stored in an Event Queue. Thus, the events are extracted by order, always getting the first one in time (which corresponds to the next simulation). On the one hand, a network simulator calculates the future events in a row when starting the simulation. This information is sent to the engine which stores the corresponding events in the Event Queue. On the other hand, MASON simulator (and Matlab/Simulink) updates the state each time step. The events corresponding to this time step $T_{step}$ are also stored in the Event Queue. At implementation stage, the value of $T_{step}$ in the social simulator must be fixed to meet REQ#4 about the tool’s accuracy. In our particular case, developers selected a value of $T_{step}=1s$ as default value, however the decision making to obtain this value it not the objective of this paper.

Once the three simulators (NS3, Matlab and MASON) have been adequately configured, they halt the simulations and only update them when informed by the third-party engine. During the co-simulator operation, the engine checks the simulator to which the first event in the Event Queue belongs. Then it removes this event from the queue and informs the corresponding simulator to continue with the next step (if the corresponding simulator is Matlab or MASON) or to execute the next event (is the corresponding simulator is NS3). The proper domain-specific simulator runs the simulation and generates certain information that could be relevant to the other simulators. The engine processes this information and sends it to the other simulators which update their information and informs the engine once done.

At the end of the simulation, all generated logs are stored in the data base.

Using this approach, moreover, REQ#3 about scalability is completely fulfilled. Simulations may take a very long time, but they admit perfectly to consider various tens of thousands of agents.

It must be noted that the previous solution it is only one of the several available possibilities. In other situations or application scenarios, the proposed scheme could not be suitable. In particular, it is also possible to create a continuous-time simulator (which updates the state each $T_{step}$ seconds) by using a different implementation of *simulator* class in the NS3 implementation or by recovering the entire simulator with an adequate interpolation middleware. The users’ preferences or the application requirements will help developers to select the most adequate approach (see Section 3.1).

On the other, although it is not mandatory, the simplest configuration for a co-simulator which includes various continuous-time domain-specific simulators is to employ a unique $T_{step}$ for every tool. Of course, the selected value of the $T_{step}$ parameter at production time will affect the correctness and validity of the obtained results. In genera words, as $T_{step}$ gets greater, the precision of the results go down. This idea is common to all continuous-time simulators, but it is especially important in co-simulation tools as many different and independent tools are involved.

Any case, these discussions must be addressed at implementation and production time (respectively) so they are not the focus of the article.

STEP #4. Design of the user interface and results presentation

In the fourth step the user interface is designed. As said in CHAR#8, the planned co-simulator is going to be limited to systems deployed in large facilities. Thus, users do not require additional instruments to design their own scenarios, and layouts of the main facilities (stadiums, colleges, etc.) may be provided with the simulator.

**Figure 13:**
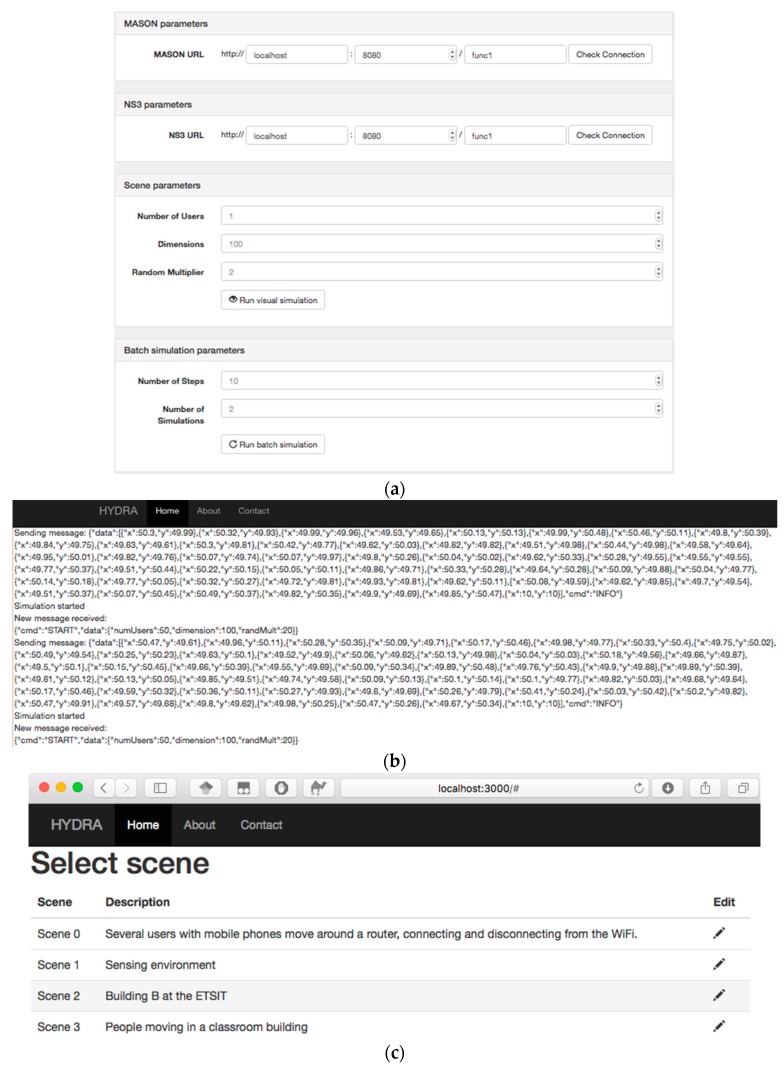

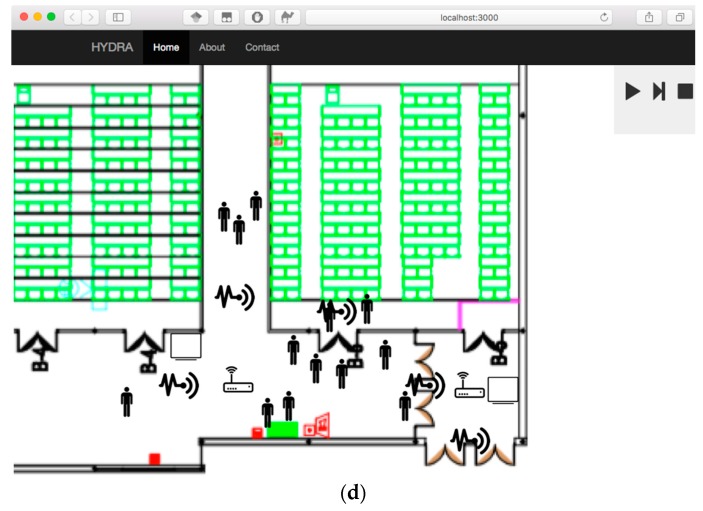
Simulator’s user interfaces. (**a**) Basic configuration. (**b**) Simulation execution and control. (**c**) Simulation design. (**d**) Results presentation (temporal animation).

As said in CHAR#2, it is unnecessary to control the evolution of the simulation step by step. The interest is focused on the global tendencies, so many simulations are performed in a row in order to obtained representative statistical results. Then, a textual interface should be included for simulation control and execution. Finally, as said in CHAR#6, different visualizations of the results have to be available. Moreover, users have to be able to calculate many important aggregated values. In conclusion, “post-mortem” results presentation is the most adequate in the proposed case. Finally, Figure 13 shows the interfaces obtained for the co-simulator.

### 4.2. Experiment Description

In order to evaluate the correctness of the proposed methodology an experimental validation was designed and carried out. The objective is verifying that the proposed methodology generates the most adequate co-simulator design for a given application, as well as the operational limits of the generated tool. In order to evaluate that, the previously designed co-simulator was implemented.

The experimental validation was divided in three different experiments.

In the first experiment (named as experiment#1) various quality parameters about the obtained co-simulator in Section 4.1 are evaluated by a crowd simulation expert. Although this first experiment pretends to be a global evaluation, the selected quality parameters are mainly focused on steps one, two and four (as well as on the final users’ requirements). Thus, the second experiment (named experiment#2) is focused on the third step. In this second experiment, temporal measurements about the required simulation time in different situations are done. Finally, in the third experiment (identified as experiment#3), the operation limits of the generated tool are evaluated. In particular, scalability and accuracy are validated.

#### 4.2.1. Non-Methodological Co-Simulator Implementation

In all the described experiments, the results are compared to the values obtained from other additional co-simulators which have been implemented without following the proposed methodology. In particular, in order to carry out the experimental validation three additional co-simulators were implemented. These new co-simulators were developed following existing proposals about this topic, instead of following the proposed methodology.

**Figure 14:**
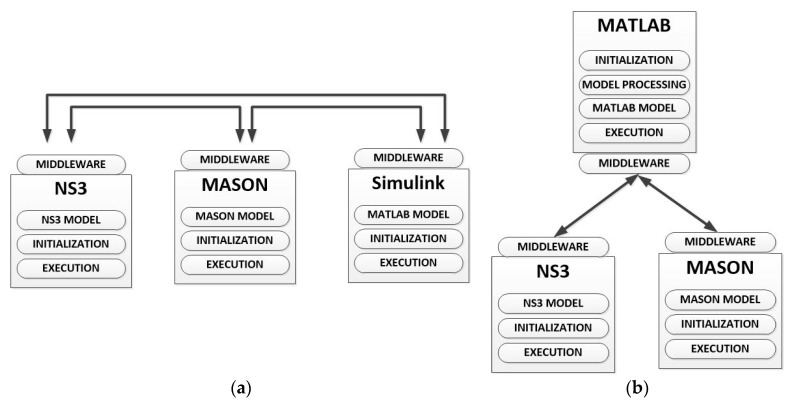
Secondary (auxiliary) co-simulators architecture. (**a**) simulator#1. (**b**) simulator#2.

The first non-methodological co-simulator (hereinafter called “simulator#1”) it was implemented following a kind of choreographed co-simulation paradigm, where every domain-specific simulator is recovered by a middleware being able to communicate with the rest of simulators [132]. Simulation models were maintained as defined by default in the domain-specific simulators, and no additional interface was deployed. All the domain-specific simulators are running in parallel. Graphics and results were shown using the instruments provided by NS3 simulator. Figure 14a describes the architecture of this new co-simulator. The simulation update process follows an iterative paradigm, where each simulation step implies a convergence phase. Every domain-specific simulator has to calculate in an independent way a first estimation of the situation of the scenario under study in the next step, which it is shared with the rest of the tools later. Considering this new information, domain-specific tools modify their initial calculations and the sharing and updating process is repeated until values converge to a stable situation.

The second additional co-simulator (hereinafter called “simulator#2”) was implemented following one of the most popular schemes for first concept proofs in research. It is similar to an “orchestrated by a one of the simulators” co-simulation paradigm, where the most versatile domain-specific tool controls and manage the entire simulation [115]. As in the base work, we have selected as main simulator the MATLAB suite. As in simulator#1, simulation models were maintained as defined by default in the domain-specific simulators, and no additional interface was deployed. A “stop-and-wait” coordination mechanism was applied and graphics and results were shown using the instruments provided by MATLAB. Figure 14b describes the architecture of this second new co-simulator.

Finally, a third additional non-methodological co-simulator was implemented. This final co-simulator (hereinafter called “simulator#3”), it is identical to the methodological one. However, in this case, a parallel execution coordination mechanism is selected, instead of the stop-an-wait mechanism employed in the methodological simulator. With this structure, this co-simulator is perfect to evaluate, in a comparative way, the performing of the selected coordination mechanism (third step in the methodology).

On the other hand, in order to allow comparisons between the proposed application-specific co-simulators and state-of-the-art tools, a benchmark simulator is also considered. As said in Section 2, no simulator proposed in the state-of-the-art allows simulating CPSS considering all subsystems in the same detail level (for example, if a fire is simulated, a realistic evolution depending the scenarios has to be followed, but also people’s behavior has to be adapted to this situation and the impact in communications should be also evaluated—for example interferences in radio channels due to smoke-). In that way, benchmark domain specific simulators use to present a better performance (as said previously, simulations are faster and more scalable) because their simulations are simpler (coordination delays and congestion, for example, do not appear). However, in order to stablish a reference, it has been selected a benchmark simulator focused on the simulation of the selected study scenario (see Section 4.2.3). In that way, the social simulator MASSIS [133,134,135] (Multi-agent system simulation of indoor scenarios) has been also deployed and employed. This simulator is based on MASON (as the proposed co-simulator), but includes new interfaces and functionalities to simulate some aspects of the cyber and physical world.

#### 4.2.2. Detailed Description of Experiments

An expert on crowd simulations was invited to evaluate the performing and adequacy of the proposed (methodological) co-simulator using a collection of quality parameters. Additionally, the same evaluation was carried out using the non-methodological co-simulators “simulator#1” and “simulator#2” (described above, Section 4.2.1) and MASSIS simulator. The list of quality parameters was selected to represented how much adequate is the obtained co-simulator for the application scenario. Moreover, the adaptation of the three co-simulators under study to the final users’ requirements is also evaluated. These parameters were: usability by crowd management experts, facility to include new types of devices, scalability to advance scenarios, adequacy of the simulation model, accuracy of the simulations, customization and interest of the presented results.

As can be seen, experiment#1 cannot evaluate which coordination mechanism (parallel execution or “stop-and-wait”) is the most adequate for our application (mainly because that is a technical decision and depends strongly on the developers being related to the co-simulator’s implementation). Then, in the second experiment, a validation was carried out in order to determine if the methodological co-simulator (Section 4.1) implements the most adequate coordination mechanism. For that, the required simulation time by the methodological co-simulator is evaluated, and compared with which required by the non-methodological co-simulator simulator#3.

Additionally, all results are compared and valuated together with the invested development time in implementing each tool. The validation consisted of the definition of various simulations which were performed using every simulator. Data about the different simulation times were registered. Finally, as we have said in Section 3.1 and Section 4.1, every designed co-simulator should fulfill the final users’ requirements. However, sometimes, as time passes, the use of the designed co-simulator gets far from what originally planned. In this case, it is very interesting to know if the new tool continuous meeting the final users’ requirements in that new context. Then, in the third experiment, three evaluations were carried out in order to determine the operational limits of the tools designed with our methodology. Moreover, result of the experiences described below were compared with results from all the implemented non-methodological co-simulators (”simulator#1”, “simulator#2” and “simulator#3”).

In this third experiment, firstly, we are evaluating the scalability of the proposed simulators, depending on the number of agents per simulation. For that, the number of agents in a certain simulation is increased until the designed co-simulator cannot execute it. For every case, various attempts are programmed. Data about the success in the simulations execution are collected. Secondly, the same evaluation is repeated for a fixed number of agents but increasing the number of parameters considered for each agent. The scalability depending on the complexity of the agent model is, thus, evaluated. As previously, for every case, various attempts are programmed. And, thirdly, it is evaluated the accuracy of the resulting co-simulator. For that, the expert on crowd simulations was asked to perform a certain simulation using independent simulation techniques. The results are taken as reference. Then, various simulations modifying the temporal step when possible are performed. Differences between these simulations and the reference (the committed error) are evaluated and employed to determine the simulator’s accuracy. As MASSIS describes entities with a fixed number of parameters, and time step is not easily modifiable, this benchmark simulator was only considered for the first part of the experiment. Although the obtained results are not comparable at all (as the complexity of the performed simulations is not similar), these measures may be employed as reference during the discussions.

#### 4.2.3. Simulation Scenario

In order to perform the described experiments, a particular simulation scenario was defined.

One of the most interesting topics nowadays is the adequacy of public infrastructures to emergency and evacuation situations (in order to avoid human avalanches, bottlenecks at the exits, etc.). In these situations, communication networks (especially proprietary WiFi networks) support a high stress and, even and depending on the situation, they may get isolated and uncommunicated. Besides, in these situations, people many times behave in a non-standard way, so regular emergency systems (such as alarms, panels, etc.) are not as much effective as desired. In these scenarios, then, the physical world (where the emergency starts and evolves), the cyber world (devices and platforms employed nowadays as main information source) and social environment (people) are totally interconnected. Thus, the purpose of the planned simulations is to test the proposed methodology in the creation of a tool for a realistic CPSS application.

**Figure 15:**
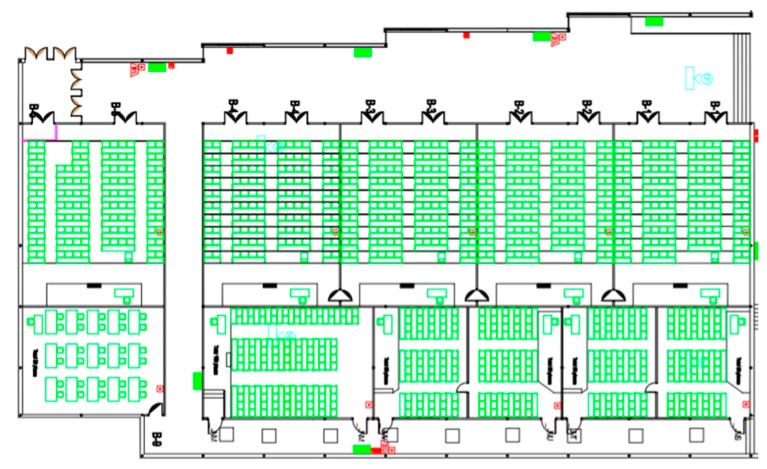
Simulated space.

The simulation scenario is a representation of first floor in one building at the Technical University of Madrid. In those spaces, we consider a certain amount of people moving around with their smartphones and interacting with the rest of people. The simulation scenario includes three corridors, ten classrooms (some of which were cooled) and other minor spaces (such as a small hall). As maximum, 800 people can be at the same time in that space. Figure 15 represents that scenario.

A fire was simulated in that scenario, where and evacuation was, then, started. The differences of temperature in the building and the information provided from the emergencies system forced people to move and interact with the others. These temperature differences cause both the people movement to change and the quality of communications to go down (properties of wireless channel change). At the same time, smartphones registered the social information and transmitted the acquired data to a central server deployed in a computer center near the simulated building (where the actions to be taken in order to manage people were calculated). With this information, groups of people may be located, specific evacuation plans and information may be communicated, etc.

The simulation pretended to calculate the system evolution along the first ten minutes.

During the first experiment (experiment#1), the expert on crowd simulations performed various simulations modifying as much aspects as he considered adequate to evaluate the proposed quality parameters. During the second experiment (experiment#2) the explained simulation was repeated considering different amounts of people in the building. In that way, it was possible to know if the difference in the development time between the methodological simulator and an identical simulator which implements parallel execution (simulator#3) justifies the use of the stop-and-wait mechanism. Finally, during the third experiment (experiment#3), simulation was repeated considering different values for the variables under study (namely, the number of agents in the scenario, the number of parameters per agent and the temporal step).

For every case, simulation was repeated fifteen (15) times and the final result was calculated as the medium value.

In order to simulate each one of the proposed sub-systems (social environment, physical world and cyber domain) libraries and state-of-the-art proposal have been employed. In particular, cellular network and WiFi elements have been simulated employing some existing NS3 libraries (WiFi and LTE modules). Social environment has been programmed using the MASON tools and proposals about emergency simulations [136]. The basic idea is to employ the inertial sensors included into intelligent terminals to determine the people behavior during the emergency (if panic is present, people keep calm, etc.). In these scenarios, participative sensing (as some works on CPSS describe) has no sense, and passive monitoring is the most adequate approach. Sensor outputs are emulated by the coordination of social simulation (which determines the evolution in the people behavior), network simulation (which control the behavior of smartphones) and physical simulation (which determines a realistic sensor output for each situation and agent). Physical domain is evaluated using numerical models for fire propagation [137].

## 5. Experimental Validation: Results

In this section results of the experiments described in Section 4.2 are presented in an ordered way.

### 5.1. First Experiment (Experiment#1): Results

Table 11 shows the conclusions of the crowd simulation expert about the quality of the presented co-simulators (the methodological one and “simulator#1” and “simulator#2”).

As can be seen, the methodological co-simulator is globally better than the non-methodological co-simulators (implemented without following the proposed methodology), and the selected benchmark simulator, for the selected application. Thus, the methodology fulfills the objective of creating the most adequate co-simulator, given a certain application of CPSS. Next, we are reviewing in detail each one of the five quality parameters.

The expert considered all co-simulators require users to know details about the cyber world, which is not desirable at all. However, in the case of the methodological co-simulator, the third-party engine covers many of the technical details which must be controlled by user in the secondary co-simulators. In relation to this point, nevertheless, MASSIS simulator is the best as it includes a graphic interface which is very useful for crowd simulation researchers.

Similar to the previous discussion, the third-party engine offers a common and easy mechanism to extend the proposed co-simulator if new devices or domains (such as the artificial world) want to be included. On the contrary, the “simulator#1” co-simulator requires to design a new middleware each time a new domain-specific co-simulator is included, which is not easy for non-technical users. The same problem appears on “simulator#2” co-simulator, as every new device requires creating an equivalent description in MATLAB language, which is most times unknown by social experts. On the other hand, MASSIS simulator offers an embedded library of available devices which is very difficult to extend (it requires to initiate a new development and to have technological knowledge).

In respect to scalability, the expert found the methodological simulator may address every simulation scenario for crowd management systems verification perfectly. This characteristic is also available in the non-methodological “simulator#2” co-simulator, as it is an intrinsic property of “stop-and-wait” coordination mechanism (as we are seeing later). On the other hand, “simulator#1” co-simulator has many problems with certain scenarios, as it must manage a great amount of signalization (typical of choreographed schemes) which hinders the simulation performing. MASSIS simulator, however, includes a heavy 3D interface which causes large scale simulations to experiment problems when a high number of elements are simulated (see Section 5.3).

The models, as the expert said, were adequate in all cases; although some improvements (such as extending the social part) could be included. Experts also indicated that simulation model included into MASSIS shows some deficiencies in respect to mobile entities (for example, they cannot correct the itinerary if a wrong path is followed).

In general, accuracy in all co-simulators is acceptable. However, non-methodological co-simulators (as well as MASSIS simulator) allow final users to control more exactly this parameter (as the third party engine hides some details), so they are more positively considered. In respect to customization, in general, the non-methodological and MASSIS co-simulators allow users to modify the models, simulation routines, etc. in an easier way than the methodological co-simulator (mainly because of the third-party engine).

Finally, the results presentation in the methodological co-simulator was very useful for crowd analyses. But the secondary co-simulators need a more extended catalogue of functionalities in this sense (for example, including advanced animations). MASSIS includes a useful graphic interface but certain representations are difficult to obtain.

In balance, as we said, the proposed methodology allowed implementing the most adequate co-simulator.

### 5.2. Second Experiment (Experiment#2): Results

**Figure 16:**
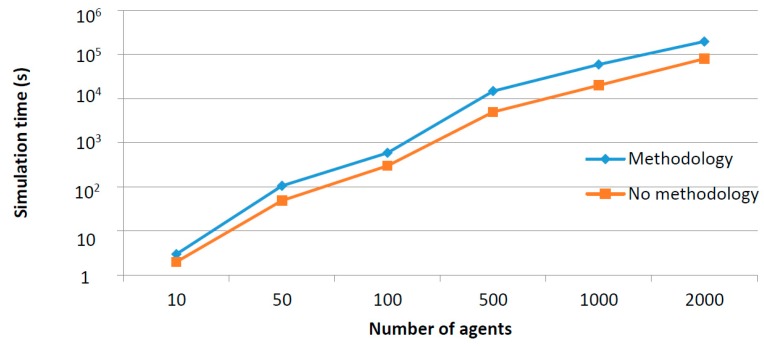
Simulation time along the number of involved people.

Figure 16 shows the results obtained from the second experiment in the experimental validation. The figure shows the average simulation time depending on the number of involved people and mobile devices (agents). In this figure, we compare the simulation time required by the methodological co-simulator, and by an identical simulator which implements parallel execution. Considering the maximum capacity of the scenario (800 people) and an over-capacity around 20%, the maximum number of agents in a simulation is two thousand (one thousand people and their corresponding smartphones).

Results on Figure 16 are presented in a logarithmic scale in order to visualize correctly all the values (despite being various magnitude order different). As can be seen, the simulation time increases for both co-simulators when increasing the number of simulated people in, approximately, a complexity of order $\sigma \left(n^{2}\right)$. As it was foreseeable, the methodological co-simulator, which implements a stop-and-wait mechanism, requires more time than the co-simulator which implements parallel execution. This difference grows with the number of agents; however, it is not enough to justify the development time required to implement the parallel execution scheme.

**Figure 17:**
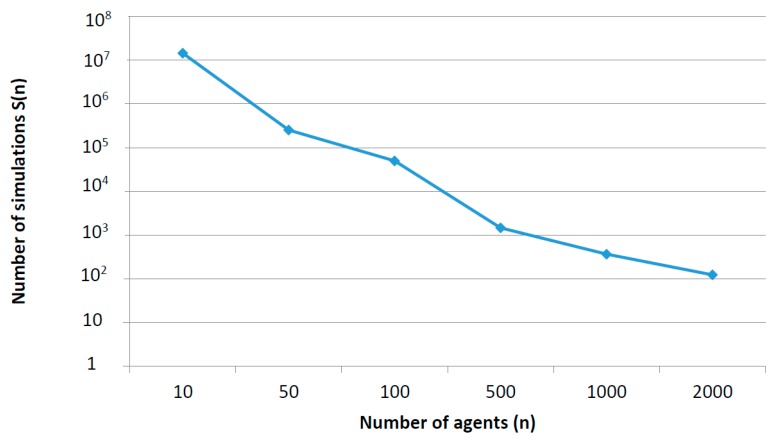
Function S(n).

In fact, we valued the total time required to implement the methodological co-simulator in one thousand (1000) h. In the case of the identical simulator which implements parallel execution, that time increases to five thousand (5000) h. With these values, in Figure 17 we represented the function S(n), taking into account the results shown in Figure 16.

As can be seen on Figure 17, in the worst case, more than one hundred (100) simulations must be performed to compensate the development time invested in implementing the parallel execution mechanism. This value grows up to ten million if the simplest scenario is considered. Any case, it is clear that the number of simulations is great, concluding that the most adequate mechanism, in this case, is stop-and-wait as indicated by our methodology.

### 5.3. Third Experiment (Experiment#3): Results

Once methodology performance has been evaluated, it is important to determine the operational limits of the co-simulator.

**Figure 18:**
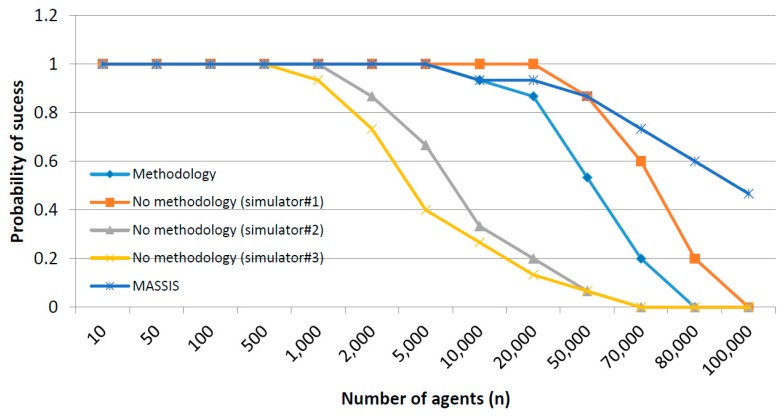
Scalability of the proposed co-simulators considering the number of agents.

Figure 18 shows the results about the scalability depending on the number of agents per simulation scenario. As can be seen, every simulation including 5000 agents, or fewer, is perfectly executed by the methodological co-simulator. However, as this number goes up, the simulation algorithm start being collapsed and, in some occasions, simulations cannot be performed. In particular, for a number of agents n=50,000 the simulation returns an execution error in 50% of times. For this work, we are considering a simulation fails (or returns an execution error) when the management entity is not able to communicate with at least one of the domain-specific simulators that make up the general co-simulator. Besides, simulations are considered blocked if the updating process in one of the domain-specific simulators does not finish before one Linux *keepalive* probe (employed, for example, also in TCP connections −75 s). In fact, problems in the simulation may appear due to hardware limitations, software coordination malfunctions or communication stack overflows because of the great amount of time required to perform the simulation update at each time step when the number of agents is above a certain limit. Furthermore, the maximum number of agents that may be considered without appearing problems in the simulation also depends on the machine hosting the simulation environment. In this case, we have selected a 64-bit Linux Ubuntu 16 operating system, with an Intel i5 processor and 8 GB of RAM memory. As this hardware configuration it is not specifically designed to support heavy simulations, obtained results may show a quite small maximum number of agents per simulation. However, as the objective of this experiment is to compare the performance of different co-simulator configurations, obtained measures can be considered adequate.

As can be seen in Figure 18, for n=80,000 or more agents the simulation algorithm never worked (in the sense expressed above). The origin of this limit is the third party engine. As this element acts as an orchestrator, all information must be processed by it. Thus, it creates a bottleneck which blocks the simulation, even when the underlying simulators may perform simulations including more agents.

This phenomenon is common to all orchestrated schemes, including the non-methodological co-simulators “simulator#2” and “simulator#3”. In the case of these simulators the problem aggravates. Fist, in “simulator#2” the orchestrator is one of the domain-specific simulators (MATLAB in this case), so this element not only must perform the orchestration activities but also the simulation under evaluation. Then, the bottleneck narrows and the maximum number of agents decreases (in this case for n=7000 agents the simulations return an error in the 50% of cases). On the other hand, “simulator#3” presents a parallel execution scheme. Thus, information is not generated in an ordered way (as in stop-and-wait scheme) and the third-party engine gets overloaded much sooner. This case is the most critical, as can be seen in Figure 18, and for n=5000 agents the simulations fail in the 60% of cases.

This situation is relieved if choreographed simulations are considered, as in “simulator#1”. It presents the widest operation regime as it presents a distributed management system. In particular, this simulator gets blocked when the signaling load among the domain-specific simulators cannot be managed. This limit is reached, approximately, for n=70,000 agents, when simulations return an error the 40% of times.

Although these numbers may seem low, greatest public facilities may host between 100 and 200 thousand people. Furthermore, the proposed simulation scenario, as maximum, may host around three thousand people (this quantity is supported without problems by the proposed co-simulator). For large-scale simulations (including hundreds of thousands of people) the designed tool is not valid but, in this case, the proposed methodology would have produced a different co-simulator. Besides, it must be considered that the employed hardware platform is not a great computational power platform as employed in other scalability studies [138]. Thus, obtained results may be slightly worse than usual.

Finally, as can be seen, benchmark simulators, such as MASSIS, allow us to perform bigger simulations without experimental problems (even the more basic MASON simulator may be configured to include near one million of agents) [138]. However, in these cases, simulations only include social phenomena, and the proposed co-simulator also offers a perspective about the state of the physical environment and the deployed technological systems.

**Figure 19:**
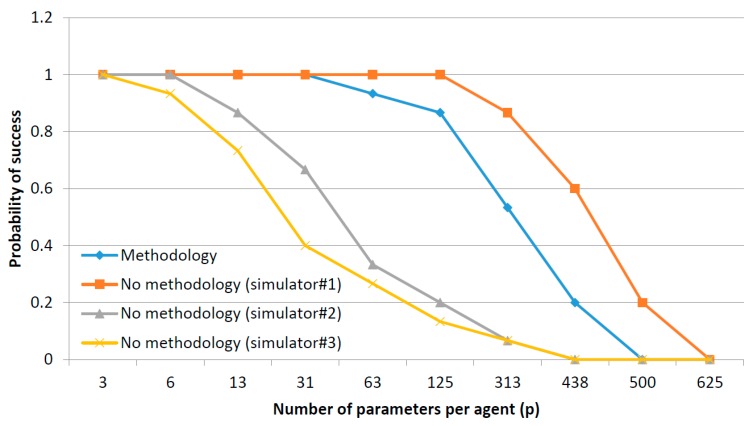
Scalability of the proposed co-simulators considering the number of parameters per agent.

As said in Section 4.2, other important variable in scalability is the number of parameters considered per agent. Figure 19 shows the obtained results. As indicated in Section 4.2, the simulations were performed including 800 agents in the scenario.

As can be seen, tendencies in Figure 19 are very similar to those observed in Figure 18. That is coherent, as simulation algorithms and signaling load depend in the same way on the number of agents and on the number of parameters per agent. Thus, discussions presented for Figure 19 are valid for this new evaluation. In order to compare the results, Table 12 presents the most important points on Figure 19.

From results on Figure 18 and Figure 19, it is possible to say that the methodological co-simulator meets the final users’ requirements. In particular, various tens of thousands (specifically between 50,000 and 80,000) may be included in a simulation scenario as indicated in REQ#3 (which specifically indicated a limit of 50,000 agents per simulation).

**Figure 20:**
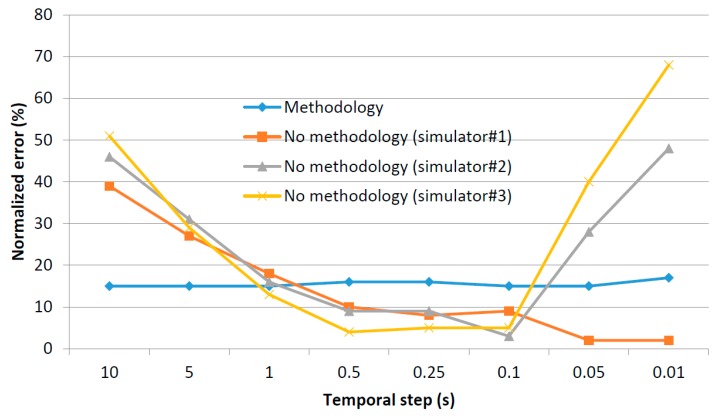
Accuracy of the proposed co-simulators.

Finally, Figure 20 shows the results for the accuracy evaluation. This evaluation considers a definition of global error (1) in order to evaluate the accuracy of the proposed co-simulator as the temporal step goes down (when possible to modify it). This expression represents the aggregated absolute error in the simulations, being $P\left(t_{final}\right)$ the final value (at the end of the simulation under study) of the parameter P and $P_{ref}\left(t_{final}\right)$ the final value (at the end of the simulation taken as reference) of the reference parameter $P_{ref}$. Results on Figure 20 are normalized.

$$\varepsilon \;=\;\underset{\forall \;agent\;A}{\sum }\underset{\forall \;parameter\;P\;in\;A}{\sum }\left|P\left(t_{final}\right)-P_{ref}\left(t_{final}\right)\right|$$

As can be seen the committed error in the proposed methodological co-simulator is constant. That is due to the fact that the temporal step is not controllable by final users (CHAR#2 showed that this functionality was not necessary). As explained in Section 4.1, the obtained co-simulator is event-oriented, so the value of the temporal step does not affect the final accuracy. The committed error is around 12% (Figure 20).

In the case of the non-methodological co-simulator all of them allow to manipulate the temporal step $T_{step}$. Thus, in general, for great values of this variable the maximum error goes up (as changes in the simulation are very abrupt), and for smaller values of $T_{step}$ the error goes down (the simulation evolves in a more continuous way). However, as can be seen, “simulator#2“and “simulator#3” do not follow this rule at all. In these simulators, the committed error goes up again when the temporal step falls below a certain limit ($T_{step}\;\approx 0.1s$ in both cases). This evolution may be explained by the fact that, in both cases, the simulation algorithm gets blocked when the temporal step is very small. In particular, the orchestration entities cannot update the global state fast enough and some information is lost. As a consequence, the error increases.

### 5.4. Discussion

Considering the previous results, the correctness of the proposed methodology may be confirmed. The results obtained from the first experiment guarantee the selected co-simulation paradigm, the designed simulation model and lifecycle and the planned results presentation interface are the most adequate. The second experiment shows the designed simulation tool is the most adequate in terms of time (the type and number of simulation performed worth the invested time in developing the tool). In general, the correctness of the third step is much more solid, as quantitative and objective proofs have been provided (second experiment). However, the rest of steps involve the users’ needs and impressions, so results are much more subjective (although a specialist has been introduced in order to reduce at minimum the human factors). For example, although scenario customization capabilities are not necessary, users prefer to have these functionalities (see Table 11).

The proposed simulator, moreover, present a wide operation range. The limiting property is the scalability, as simulations including more than 80,000 agents (or more than 500 parameters per agent) cannot be performed. Any case, final users’ requirements are perfectly met.

As said, the proposed methodology is correct as it generates the most adequate tool for each application (especially in terms of costs and time). However, the possibilities of future extensions or new versions are not considered and some synergies can remain hidden when applied the proposed methodology. Any case, in these situations the most adequate simulator for a certain application it is not generated, as business or corporative arguments are the main factor to be considered.

Finally, the proposed methodology it is only the first stage in the simulator creation process, as (once defined the architecture) implementation tasks must be initiated and, finally, users should know how to perform valid simulations. These posterior phases introduce new variables, challenges and problems which are not the focus of the article but which influence the final obtained tool.

## 6. Conclusions

In the present paper, a new methodology to design efficient application-specific CPSS co-simulator has been described. The methodology includes four points which allow selecting the most adequate co-simulation paradigm, to particularize the general simulation model and lifecycle, to choose the better coordination mechanism and, finally, to design the proper interface to the particular application. Additionally, a previous phase for capturing the final users’ requirements and characteristics is described. The obtained inputs are employed as selecting criteria in the methodology steps.

Traditional works on co-simulation are focused on very particular aspects about the implementation process, or they only consider the design of integrated co-simulators, which are not the most adequate for all applications. On the contrary, our proposal enables users to design (and implement, if desired) the co-simulator most appropriate for their applications.

First evidences that the simulators generated using the proposed methodology are the friendliest and most useful for users and experts on a certain application of CPSS are provided, as indicated for crowd experts during the proposed experiments. Moreover, the methodology also tries to be efficient in time, taking into account and balancing both elements: the required simulation time and the needed development effort. In that sense, solid evidences that our proposal helps users to select the best option among all the possibilities for a certain application, following an ordered process have been provided. Nevertheless, further analyses considering the collaboration of CPSS researchers should be performed in order to determine the final usefulness of the proposed methodology.

Future works should address the problems mentioned above, organizing deep and large-scale experimental studies involving CPSS researchers. These professionals, who should need to design a new co-simulator, would report their experience, which could be compared with data from a control group. Besides, the relation between the proposed design phase and the next stage (usually, implementation) should be analyzed in order to improve some aspects of the proposed solution (for example, enabling, in that way, the use of agile methodologies, very common in software development). Moreover, and finally, the inclusion of automatic tools in order to build valid simulation models, adequate for the designed co-simulator should be also considered.

## Figures and Tables

**Table 1:** Different concepts about the relations among the CPSS subsystems.

Concept	Physical World	Social World	Cyber World
Cyber-Physical systems	x		x
Social Internet-of-Things		x	x
Social Environment	x	x	
Social sensing		x	x
CPSS	x	x	x

**Table 2:** Physical processes simulators.

Characteristics	Traditional Research Simulators	Current Commercial Simulators	Current Research Simulators	
			Mixed Simulators	Most Common Current CPS Simulators
Include a graphic environment	No	Yes	Yes	Sometimes
Domain-specific knowledge is required	Yes	No	Yes	Yes
Include aspects of the cyber world	No	No	Yes	Yes
Is a stable version	Yes	Yes	No	No
Simulation scheme	First, all the simulation are performed and, later, results are showed			

**Table 3:** Social simulators comparison.

Characteristics	Social-Level Simulators	Agent-Based Simulators		
		Ambient Intelligence	Development of IoT Systems	Generic
Represent each people as a unit	No	Yes	Yes	Yes
Programming knowledge is required	No	Sometimes	Sometimes	Sometimes
A graphic interface is provided	Yes	Yes	Yes	Yes
Cyber elements may be included	No	Yes	Yes	No
People is the objective of the simulations	Yes	Yes	No	Yes
Stable tools are available	Yes	Yes	Yes	Yes
Simulation scheme	Results are showed as calculated in each time step			

**Table 4:** Network or IT simulators comparison.

Characteristics	Traditional Network Simulators	IoT Simulators	Social IoT Simulators
Include a graphic environment	Sometimes	Yes	Yes
Programming abilities are required	Yes	No	No
Include social aspects	No	No	No
Is a stable version	Yes	Sometimes	No
Number of customizing options	High	Medium	Low
Simulation scheme	Event-driven, offering the logs in each step		

**Table 5:** Relevant characteristic for a co-simulator.

Thematic Block	Relevant Characteristics
Application characteristics	The main subsystem (physical, cyber or social world) in the simulation Type of application (e.g., Ambient Intelligence validation or development) Possible simulation scenarios considered (buildings, cities, large facilities, etc.)
Development team characteristics	Knowledge about simulators programming Number of developers (available workforce)
Final users characteristics	Technical and programming skills Users’ profile (sector professionals, researchers or students, etc.)

**Table 6:** Criteria for the selection of a co-simulation paradigm.

Group	Criteria	Explanation
Developers‘ knowledge and users‘ abilities	Technical capacity to perform large developments (programming to modify the simulators themselves)	Developing an integrated co-simulator requires great knowledge about programming and expert people on software development. If these resources are not available, federated co-simulators are preferable
	Technical skills of users (programming to implement the models)	If users have technical skills, they can perform the scenario division into different domains. If that is not possible, orchestrated or integrated co-simulation are the only feasible paradigms. These paradigms, moreover, help to consider a high number of different subsystems in the simulation without complicating the usability in excess.
Characteristic of the selected simulation tools	Utilization of open architecture tools	If simulation tools present an open architecture, federated co-simulation may be employed. However, if all selected simulators are close architecture tools, any information could be exchanged and integrated co-simulation is the solution.
	Availability of adequate domain-specific simulator in the state of the art	If any of the available domain-specific simulators nowadays is adequate to be integrated in the new co-simulator, integrated co-simulator is the only valid paradigm (programming the unavailable modules).
	Utilization of open source tools	If open architecture tools are used, and federated co-simulation is going to be employed, the use of choreographed co-simulation requires all simulators involved to be open source (as the code has to be slightly modified).
	Compatibility among the domain-specific tools	If open architecture tools are used, and federated co-simulation is going to be employed, the use of choreographed co-simulation requires all simulators involved to be totally compatible (use the same data formats, communication protocols, APIs, etc.).
Other	Results presentation and user interaction	If orchestrated co-simulation is being performed, and if none of the modules for results presentation provided with the domain-specific simulation tools meet the needs of the new co-simulator, a third-party engine must be included

**Table 7:** Criteria for the creation of the simulation model.

Application	Actions
Validation of ambient intelligence systems [91,92,93,94,122]	A more specific definition of the physical world (ambient) is necessary. In particular, physical laws for the evolution of relevant phenomena should be modeled. Besides different types of people should be also considered (depending on if they present special needs, incapacities, etc.).
Development of IoT systems [95]	The concept of “service” should be added in the simulation model, and a more exhaustive description of the different types of devices also would be desirable (in particular, a description of the interfaces is very important in IoT scenarios).
Social research [123,124]	Most elements in the cyber world can be removed (it is enough to include the concept of “device”). On the other hand, social world must be extended, including different personal and social states, different types of interrelations among people and the social evolution laws.
Crowd management [125,126,127]	A detailed model for “physical object” may be important. Models for walls, doors, buildings, etc. are critical in order to manage people in the most adequate way. In addition, models for the different emotions and their propagation in crowds must be considered.
Social sensing [86,87,88,89,90]	Different types of sensors have to be considered, so the model must include all of them. Besides, the social world requires a more exhaustive description as mentioned in the case of “social research” and “crowd management”.

**Table 8:** Coordination mechanisms.

Coordination Mechanism	Co-Simulation Paradigm	Implementation
Parallel execution	All	Every tool executes in a separate host, processor or thread. Simulation calculations are performed in parallel and results are shared with the rest of simulators immediately (using the orchestrator element if necessary).
Stops and waits execution	Choreographed	In a certain order, every simulator makes its calculations. When all tools have performed their execution, all of them share the results with the others.
	Orchestrated by a third-party engine	In a certain order, the engine order every simulator to execute the calculations. When each simulator finishes, it sends the results to the engine and it sends the information to the other tools.
	Orchestrated by one of the simulators	First, the orchestrator simulator performs its calculations and shares the results with the other tools. Then, in a certain order, it orders every simulator to execute its calculations. When each simulator finishes, it sends the results to the orchestrator and it sends the information to the other tools

**Table 9:** Criteria for designing the user interface.

Tasks	Criteria
Design a simulation	If simulations concern only a limited collection of scenarios, predefined layouts are the appropriate solution. If users must be enabled to design their own scenarios, additional external instruments are required (for example 3D modeling, development environments, etc.).
Execute and control the simulation	Text interfaces are adequate for users who performs many simulations in a row (such as in Monte-Carlo simulations). In didactic applications, or if simulations are performing one-by-one, graphical interfaces are desirable.
Analyze the results	Simulators which generates great amounts of data require “post-mortem” tools as no enough time is available to process and presents the results when performing the simulation. If the monitored variables are few (such as, for example, the position of the agents), then “live” tools are valid.

**Table 10:** User interface key elements.

Co-Simulation Paradigm	Key Elements
Independent	Individual controls Individual results
Choreographed	Global scenario definition Relationship conditions
Orchestrated by a third-party engine	Third party engine connection status
Orchestrated by one of the simulators	Main simulator selector

**Table 11:** Results about the quality parameters in the first experiment.

Quality Parameter	Marks (0–10)			
	Methodological Co-Simulator	Non-Methodological simulator#1	Non-Methodological simulator#2	MASSIS
Usability by crowd management experts	8	5	6	9
Facility to include new types of devices	9	7	6	6
Scalability to advance scenarios	9	6	9	7
Adequacy of the simulation model	8	8	8	7
Accuracy of the simulations	7	8	8	8
Customization	6	8	8	7
Interest of the presented results	9	7	7	8
**Total**	**8**	**7**	**7.4**	**7.5**

**Table 12:** Scalability of the proposed co-simulators considering the number of parameters per agent.

Important Points	Methodological	“simulator#1”	“simulator#2”	“simulator#3”
Maximum number of parameters without fails	30	300	7	4
Number of parameters 50% of fails	310	440	40	30
Maximum number of parameters	500	620	440	440

## Abbreviations

The following abbreviations are used in this manuscript:

CPSS	Cyber-Physical Social Sensing
CPS	Cyber-Physical Systems
